# Predicting patient decompensation from continuous physiologic monitoring in the emergency department

**DOI:** 10.1038/s41746-023-00803-0

**Published:** 2023-04-04

**Authors:** Sameer Sundrani, Julie Chen, Boyang Tom Jin, Zahra Shakeri Hossein Abad, Pranav Rajpurkar, David Kim

**Affiliations:** 1 School of Medicine, Vanderbilt University, Nashville, TN USA; 2 Department of Computer Science, Stanford University, Stanford, CA USA; 3 Dalla Lana School of Public Health, University of Toronto, Toronto, ON Canada; 4 Department of Biomedical Informatics, Harvard Medical School, Boston, MA USA; 5 Department of Emergency Medicine, Stanford University, Stanford, CA USA

**Keywords:** Predictive medicine, Cardiovascular biology, Predictive markers

## Abstract

Anticipation of clinical decompensation is essential for effective emergency and critical care. In this study, we develop a multimodal machine learning approach to predict the onset of new vital sign abnormalities (tachycardia, hypotension, hypoxia) in ED patients with normal initial vital signs. Our method combines standard triage data (vital signs, demographics, chief complaint) with features derived from a brief period of continuous physiologic monitoring, extracted via both conventional signal processing and transformer-based deep learning on ECG and PPG waveforms. We study 19,847 adult ED visits, divided into training (75%), validation (12.5%), and a chronologically sequential held-out test set (12.5%). The best-performing models use a combination of engineered and transformer-derived features, predicting in a 90-minute window new tachycardia with AUROC of 0.836 (95% CI, 0.800-0.870), new hypotension with AUROC 0.802 (95% CI, 0.747–0.856), and new hypoxia with AUROC 0.713 (95% CI, 0.680-0.745), in all cases significantly outperforming models using only standard triage data. Salient features include vital sign trends, PPG perfusion index, and ECG waveforms. This approach could improve the triage of apparently stable patients and be applied continuously for the prediction of near-term clinical deterioration.

## Introduction

Triaging emergency department (ED) patients to timely and appropriate care is essential for clinical and operational outcomes. Early warning scores at triage have shown moderate success in predicting physiologic decompensation (deterioration of one or more vital signs such as heart rate, oxygen saturation, or blood pressure) and mortality^1,2^. Such scores include vital signs, assessed once at presentation, and sometimes again at variable intervals^3^. Patients with vital sign abnormalities are prioritized to a higher level of care. Standardized approaches for risk-stratifying and managing patients with conditions such as sepsis^4^, stroke^5^, cardiac arrest^6^, or chronic obstructive pulmonary disease^7^ have been well established^8^.

For patients presenting without initial physiologic abnormalities, there is no standard framework for predicting subsequent decompensation or care needs^8–10^, and unexpected clinical decompensation can arise. Some studies have shown that up to 14.5% of ED patients experience clinical decompensation^11^, with up to 12.9% experiencing unreported decompensation (i.e., development of abnormal vital signs without clinician notification), particularly in overcrowded EDs and among elderly patients^12^.

ED patients are routinely connected to continuous physiologic monitors, which measure vital signs continuously (heart rate, respiratory rate, oxygen saturation) or intermittently (blood pressure by sphygmomanometry), as well as high-resolution electrocardiogram (ECG) and photoplethysmography (PPG) signals. Such monitors offer detailed, real-time data for potential predictive systems. Trends in vital signs and ECG/PPG waveforms may contain information about risk of deterioration not captured by a single waveform or set of vital signs^13–16^. Machine learning methods applied to multimodal bedside monitor data might therefore be used to improve predictions of clinical decompensation.

Previous work has applied machine learning to vital signs and other patient features to predict clinical and operational outcomes in the ED, such as COVID-related complications^17^, sepsis^18^, and need for hospital or intensive care unit (ICU) admission^9,19–21^. Recent research also suggests that features extracted from physiologic waveforms such as arterial blood pressure, ECG and PPG can assist in predicting vital signs^22–25^ and the development of vital sign abnormalities such as tachycardia^25,26^, hypotension^27–29^, hypoxia^30,31^ or death^32^. Prior work has analyzed physiologic waveforms to predict specific clinical events such as atrial fibrillation within 45 minutes^33^, blood pressure response to fluid administration within 3 hours^34^, hemodynamic decompensation in simulated hemorrhage patients^35^, and fluid shifts during hemodialysis^36^. Most prior studies including physiologic waveforms are conducted in ICU, operating room, or laboratory settings. To our knowledge, no prior study has combined continuous numeric vital signs and physiologic waveforms to predict decompensation in a general ED population, whose underlying diagnoses and disease severity are often not established, or are actively evolving, at the time of initial presentation.

In this study, we predict the clinical decompensation of initially stable ED patients using multi-modal physiologic data from the first 15 min of monitoring. Specifically, we develop *VitalML*, a multimodal machine learning framework that learns patient physiology through both engineered features and deep learning-derived ECG/PPG waveform embeddings, to predict which patients will develop tachycardia, hypotension, or hypoxia in the next 90 min. We also predict critical values of a validated composite measure for patient decompensation, the Modified Early Warning Score (MEWS)^37^. We characterize the features most relevant to each prediction and conclude with clinical implications for patient triage and monitoring.

## Results

### Study overview

**Fig. 1:**
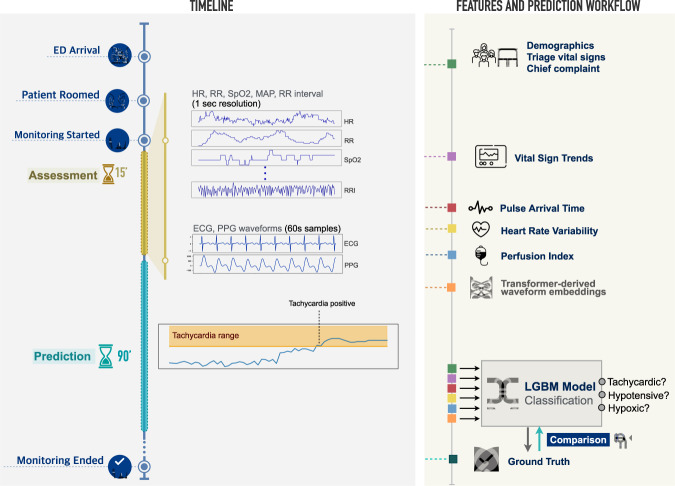
Data sources and modeling approach. Patient demographics, chief complaint, and initial vital signs are collected upon ED arrival. After rooming, and concurrent with other workup, the patient enters a 15-minute assessment period during which six numeric measures (HR, RR, SpO2, MAP, Beat-to-Beat RR Interval, Perfusion Index) are recorded at 1-second resolution, and 60-second segments of lead-II ECG and PPG waveforms are sampled. Triage data and vital sign trends are combined with physiologic measures derived from RR intervals and ECG/PPG waveforms (heart rate variability, pulse arrival time), as well as deep learning-derived representations of ECG and PPG waveforms, in a model that predicts whether a patient will develop tachycardia, hypotension, or hypoxia in the 90 min following the initial assessment period.

We developed *VitalML*, a multimodal machine learning framework using data from physiologic monitors to identify initially stable patients at risk for clinical decompensation (tachycardia, hypoxia, or hypotension) within 90 min of initial assessment (Fig. 1). Our approach extracts features from continuous ECG and PPG waveforms, using both conventional signal processing techniques to extract features of known clinical relevance (heart rate variability and pulse arrival time), as well as embeddings derived from transformer-based deep learning models.

We assessed four classes of features in the prediction of clinical deterioration: features observed at ED triage, features recorded directly by bedside monitors during initial monitoring, features engineered from ECG and PPG waveforms, and deep learning-derived embedding representations of ECG and PPG waveforms. Triage features included patient age, gender, Emergency Severity Index (ESI) assigned at triage, vital signs at triage (HR, RR, SpO2, MAP, SBP, DBP), and 46 indicator variables for categories of chief complaint at triage. Directly monitored features included first vital signs during the assessment period (HR, RR, SpO2, MAP, SBP, DBP), and the coefficients of linear trends in these features during the assessment period. Features engineered from ECG and PPG waveforms included several measures of heart rate variability (HRV) derived from beat-to-beat RR interval and from the ECG waveform itself, and pulse arrival time (PAT) as measured from concurrent ECG and PPG waveforms. Finally, we used transformers to generate waveform embeddings from 60-second ECG and PPG samples.

### Visit characteristics

We studied 19,847 adult ED visits to monitored beds with normal vital signs (HR ≤ 110bpm, SpO2 ≥ 90%, and MAP ≥ 65 mmHg) at triage and during the first 15 min of monitoring (the assessment period). In the 90 min following the assessment period, new tachycardia (HR > 110bpm) developed in 6.11% (1213/19,847) of visits, hypoxia (SpO2 < 90%) in 11.20% (2222/19,847), and hypotension (MAP < 65 mmHg) in 2.33% (462/19,847). For each outcome, patients experiencing vital sign abnormalities were significantly more likely to be admitted to the hospital. Visit details are described in Table 1.

**Table 1:** Characteristics of visits for each prediction cohort.

Characteristic	Tachycardia			Hypoxia			Hypotension		
	Decomp. (*n* = 1213)	No Decomp. (*n* = 18634)	*p* value of diff.	Decomp. (*n* = 2222)	No Decomp. (*n* = 17625)	*p* value of diff.	Decomp. (*n* = 462)	No Decomp. (*n* = 19385)	*p* value of diff.
Age in years, median [IQR]	53.0 [35.0–70.0]	61.0 [44.0–75.0]	<0.001	65.0 [50.0–79.0]	60.0 [43.0–75.0]	<0.001	64.0 [39.25–77.75]	61.0 [43.0–75.0]	0.405
Female, *n* (%)	628 (51.77)	9491 (50.95)	0.578	1135 (51.08)	8984 (50.99)	0.934	275 (59.52)	9844 (50.79)	<0.001
Male, *n* (%)	585 (48.23)	9138 (49.05)		1087 (48.92)	8636 (49.01)		187 (40.48)	9536 (49.21)	
Triage VS, median [IQR]									
SpO2, %	99.0 [97.0–100.0]	99.0 [97.0–100.0]	0.090	98.0 [97.0–100.0]	99.0 [98.0–100.0]	<0.001	99.0 [97.0–100.0]	99.0 [97.0–100.0]	0.010
Resp. Rate	18.0 [16.0–20.0]	18.0 [16.0–19.0]	<0.001	18.0 [16.0–20.0]	18.0 [16.0–19.0]	<0.001	18.0 [16.0–20.0]	18.0 [16.0–19.0]	0.800
Heart Rate	97.0 [88.0–103.0]	81.0 [72.0–91.0]	<0.001	83.0 [72.0–93.0]	82.0 [72.0–92.0]	0.052	81.0 [70.0–92.0]	82.0 [72.0–92.0]	0.180
Systolic BP	137.0 [122.0–152.0]	138.0 [123.0–153.0]	0.193	135.0 [120.0–151.0]	138.0 [123.0–153.0]	<0.001	116.5 [105.0–132.0]	138.0 [123.0–153.0]	<0.001
Diastolic BP	83.0 [73.0–93.0]	79.0 [69.0–89.0]	<0.001	78.0 [68.0–89.0]	79.0 [69.0–89.0]	0.104	66.0 [58.0–79.75]	79.0 [69.0–89.0]	<0.001
MAP	101.0 [91.0–111.3]	98.7 [88.3–109.0]	<0.001	97.7 [86.7–108.7]	99.0 [89.0–109.3]	<0.001	83.3 [75.0–96.3]	99.0 [89.0–109.3]	<0.001
ESI, *n* (%)									
Level 1	16 (1.32)	143 (0.77)	0.037	21 (0.95)	138 (0.78)	0.419	17 (3.68)	142 (0.73)	<0.001
Level 2	450 (37.1)	5481 (29.41)	<0.001	729 (32.81)	5202 (29.51)	0.001	180 (38.96)	5751 (29.67)	<0.001
Level 3	714 (58.86)	12617 (67.71)	<0.001	1422 (64.0)	11909 (67.57)	<0.001	258 (55.84)	13073 (67.44)	<0.001
Level 4	23 (1.9)	287 (1.54)	0.333	34 (1.53)	276 (1.57)	0.898	5 (1.08)	305 (1.57)	0.400
Level 5	1 (0.08)	11 (0.06)	0.748	4 (0.18)	8 (0.05)	0.015	0 (0.0)	12 (0.06)	0.593
Visit duration, hours [IQR]	6.00 [4.42–8.07]	5.65 [4.12–7.58]	<0.001	6.18 [4.57–8.1]	5.6 [4.08–7.55]	<0.001	5.88 [4.35–7.95]	5.67 [4.12–7.6]	0.029
Admitted, *n* (%)	595 (49.05)	7452 (39.99)	<0.001	1085 (48.83)	6962 (39.50)	<0.001	251 (54.33)	7796 (40.22)	<0.001
ICU, *n* (%)	36 (2.97)	265 (1.42)	<0.001	43 (1.94)	258 (1.46)	0.105	31 (6.71)	270 (1.39)	<0.001

Decompensation refers to the development of a new vital sign abnormality in the 90 min following initial assessment. Differences were evaluated with Wilcoxon rank-sum tests for numeric variables, and chi-squared tests for categorical variables.

### Prediction of new tachycardia, hypotension, and hypoxia

**Fig. 2:**
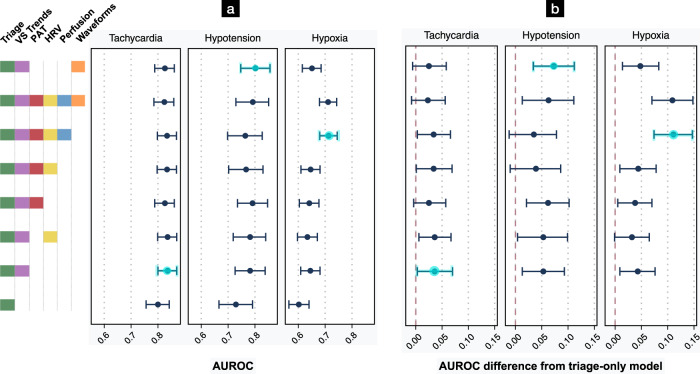
Effect of feature types on AUROC for prediction of decompensation. **a** AUROC point estimates with bootstrapped 95% CIs represent prediction performance on the test set (see also Supplementary Tables 1-2). **b** AUROC differences from the baseline triage model with 95% CIs. For each outcome, additional monitoring features produced more accurate predictions than the baseline triage model. “Triage” variables include age, gender, triage vital signs, and chief complaint. “VS Trends” denotes first vital signs from continuous monitoring, and the linear trend of each vital sign over a 15-minute assessment period. “PAT” denotes pulse arrival time, calculated from the ECG and PPG waveforms. “HRV” is a suite of heart rate variability measures. “Perfusion” is the perfusion index. “Waveforms” indicates an 8-dimensional embedding generated from a transformer model, with 4 features each from the PPG and ECG waveforms. The best-performing model for each outcome is highlighted in light blue.

The best-performing models, using both conventional triage features (age, gender, triage vital signs, Emergency Severity Index [ESI], and chief complaint [CC]), as well as features derived from a 15-minute period of continuous monitoring, predicted new tachycardia with AUROC of 0.836 (95% CI, 0.800–0.870), new hypotension with AUROC of 0.802 (95% CI, 0.747–0.856), and new hypoxia with AUROC of 0.713 (95% CI, 0.680–0.745) in a held-out test set of visits chronologically following those used in training and validation (Fig. 2, Supplementary Tables 1-2). Each model significantly outperformed the best models using only conventional triage features, with absolute AUROC improvements of +0.036 (95% CI, 0.003–0.070) for tachycardia, +0.073 (95% CI, 0.034–0.112) for hypotension, and +0.111 (95% CI, 0.074–0.147) for the prediction of new hypoxia.

### Effect of feature types on prediction performance

In the prediction of tachycardia, we observed significant improvements over the baseline model with models including vital sign trends from the first 15 min of monitoring, HRV measures, PAT, and perfusion index. Prediction of hypotension benefited from inclusion of vital sign trends over the assessment period, as well as PAT, HRV, perfusion index, and deep learning-derived waveform features. Predictions of new hypoxia were improved with inclusion of vital sign trends over the assessment period, PAT, HRV, and perfusion index (Fig. 2, Supplementary Tables 1–2).

### Prediction test characteristics

We calculated test characteristics (sensitivity, specificity, negative predictive value [NPV], positive predictive value [PPV]) for the best-performing and baseline models, selecting operating points for 0.85 sensitivity in the validation set, and evaluating performance in the held-out test set (Supplementary Tables 3-4). For prediction of tachycardia, the additional features dramatically improved model specificity, from 0.608 (95% CI, 0.588–0.627) in the best triage model, to 0.740 (95% CI, 0.723–0.758) in the best overall model. In predicting hypoxia, the best model likewise exhibited a large improvement in specificity, from 0.239 (95% CI, 0.221–0.257) to 0.365 (95% CI, 0.344-0.385). For the comparatively rarer outcome of hypotension, by contrast, the primary benefit of additional features was to model sensitivity, which improved from 0.661 (95% CI, 0.543–0.778) to 0.742 (95% CI, 0.633–0.848). Supplementary Table 3 presents detailed test characteristics at additional model operating points.

### Prediction performance for 60- and 120-min windows

We trained analogous models, and performed similar analyses, for the prediction of decompensation in the 60- (Supplementary Tables 5–7) and 120-minute (Supplementary Tables 8–10) periods following initial assessment. In the 60-minute prediction window, prediction of hypoxia was significantly improved in a full-featured model using all variable types, with +0.085 (95% CI, 0.041-0.129) improvement in AUROC over the triage model (Supplementary Table 6). In the 120-minute prediction window, models using additional monitoring features improved significantly over the baseline for all three tasks, with AUROC improvements of +0.043 (95% CI, 0.015-0.072) for tachycardia, +0.060 (0.015-0.104) for hypotension, and +0.079 (0.046–0.113) for hypoxia (Supplementary Table 9).

### Prediction of elevated MEWS score

As an auxiliary outcome, we trained analogous models to predict newly elevated values of the Modified Early Warning Score (MEWS), a composite measure of physiologic abnormalities previously validated for the prediction of decompensation and adverse outcomes^37^. Our prediction cohort (patients presenting without tachycardia, hypoxia, or hypotension upon triage and initial rooming) had correspondingly low MEWS values on presentation (median 1, IQR 1-1). We predicted whether these patients would subsequently develop MEWS ≥ 4, a threshold previously associated with increased care needs^38^. In the 90-minute window, the best-performing model used vital sign trends during the assessment period, PAT, and HRV to predict the development of MEWS ≥ 4 with AUROC of 0.825 (95% CI, 0.794–0.856), a + 0.053 (0.027–0.079) improvement over a baseline model using triage features alone (Supplementary Table 11). Supplementary Tables 11-12 show effects of feature combinations on AUROC and AUPRC for the prediction of MEWS ≥ 4 in the 60-, 90-, and 120-minute windows following initial assessment. Supplementary Table 13 shows the correspondence between predictions of specific abnormalities (tachycardia, hypotension, hypoxia), and the maximum MEWS recorded in the 90-minute prediction window.

Supplementary Figure 1 shows calibration plots for triage-only baseline and best-performing models for each outcome (tachycardia, hypotension, hypoxia, MEWS ≥ 4), in the 90-minute prediction window, as well as the result of isotonic regression fit on the validation set for the best-performing models. For prediction of tachycardia, hypoxia, and MEWS ≥ 4, the best-performing models exhibited better calibration than the triage-only baseline models, which tended to overpredict decompensation for low-risk visits and underpredict decompensation for higher-risk visits. In prediction of new hypotension, both baseline and best-performing models underpredicted the outcome at all risk levels. Isotonic regression, fit on the validation set, improved model calibration, particularly for hypotension and hypoxia, though hypotension remained under-predicted for some visits.

### Interpreting model performance

#### Identifying features with high contributions to final prediction

**Fig. 3:**
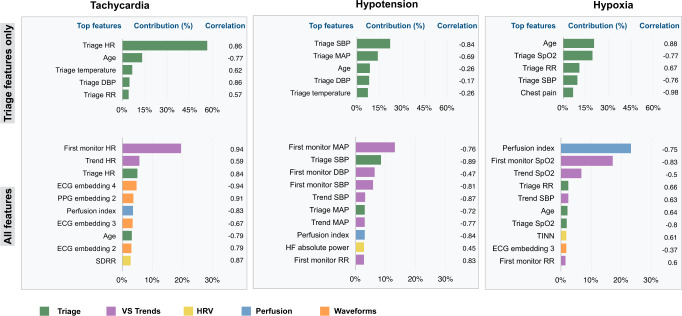
Contribution of feature types to model predictions. Mean SHAP importance values were calculated for each feature in the baseline triage-only and fully featured models for all three tasks (see also Supplementary Table 11). Contribution (%) represents the relative weight of a given feature in the model’s prediction. Pearson’s correlation coefficients represent the extent to which a higher feature value contributes to a positive prediction as assessed through SHAP analysis.

We used SHAP analysis to identify the most important features for each prediction, for the baseline and best-performing models (Fig. 3, Supplementary Table 14). We calculated correlations between SHAP scores and feature values to assess the direction of the contribution, where a positive correlation indicates that greater values of a feature contribute toward a positive prediction. Baseline (triage) models were restricted to age, gender, chief complaint, and vital signs at triage. For tachycardia, triage features associated with a positive prediction included higher HR, temperature, and diastolic blood pressure at triage, and younger age. Prediction of hypotension was related to lower blood pressure and temperature at triage, and younger age. Predictions of hypoxia were associated with increased age, lower SpO2 and systolic blood pressure, and higher RR at triage.

Given the similar performance of fully featured and best-performing models for each outcome, and the variance in best-performing models among the 60-, 90-, and 120-minute prediction windows (Supplementary Tables 1, 6, 9), we applied SHAP analysis to the fully featured models to assess the relative importance of all features. In addition to triage features, unrestricted models had access to a 15-minute assessment period of continuous monitoring, PAT, HRV, perfusion index, and deep-learning-derived waveform embeddings. These unrestricted models relied substantially on vital sign trends during the assessment period (HR for tachycardia, blood pressure and RR for hypotension, SpO2 and SBP for hypoxia), on deep-learning derived ECG and PPG waveform embeddings (tachycardia and hypoxia prediction), and on various measures of HRV and the perfusion index of the PPG waveform (the ratio of pulsatile to non-pulsatile blood flow^39^) for all three tasks (Fig. 3, Supplementary Table 14).

#### Characterizing patient populations with improved predictions in the best-performing model

**Fig. 4:**
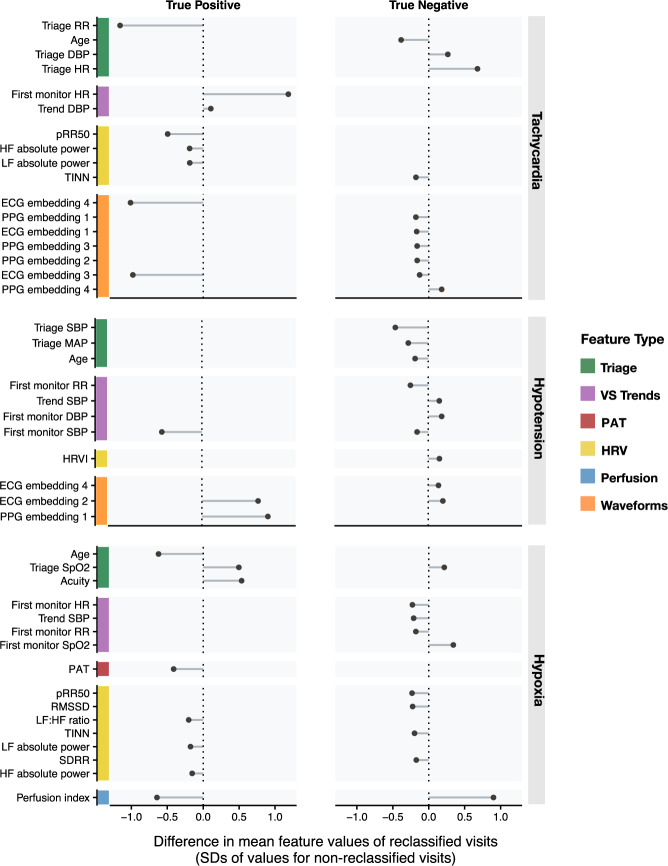
Differences in feature values among correctly reclassified visits. We isolated the visit cohorts correctly classified by the best-performing model, but incorrectly classified by the baseline triage model. For each reclassified cohort, we identified the features that differed significantly from the non-reclassified cohorts (*p* < 0.05, two-sided t-tests). Points represent differences in mean feature values for correctly reclassified visits, scaled to each variable’s distribution among non-reclassified visits.

We characterized features of visits classified correctly by the best-performing models and incorrectly by the baseline triage models to determine which patient populations benefit from more sophisticated predictive methods (Fig. 4, Supplementary Table 15). For tachycardia, patients correctly reclassified to a negative prediction had higher HR at triage, were younger, had a higher diastolic blood pressure, distinct ECG and PPG waveform embeddings, and different values of one HRV metric (TINN). Patients correctly reclassified to a positive prediction of tachycardia had distinct HRV characteristics and PPG/ECG embeddings, as well as differences in monitored HR and DBP during the assessment period. For hypotension, patients correctly reclassified to negative by the best-performing model had lower MAP and systolic BP at triage, lower RR and higher diastolic BP on first monitoring, were younger, and had distinct ECG waveform embeddings. Patients correctly reclassified to positive hypotension had distinct ECG and PPG waveform embeddings, and lower systolic BP on first monitoring. For hypoxia, patients benefiting from improved predictions in the best-performing model had extreme values of the perfusion index, different HRV profiles, and distinct vital signs during the assessment period, compared to non-reclassified visits.

## Discussion

We present *VitalML*, a multimodal machine-learning framework that uses continuous physiologic monitoring to identify initially stable ED patients who will subsequently develop tachycardia, hypotension, or hypoxia. For each outcome, we find that models incorporating features from a 15-minute period of passive monitoring significantly outperform models restricted to conventional triage features. For some outcomes and prediction windows, engineered and learned waveform features improve discrimination over vital sign trends alone. We propose that this approach could be used to improve the triage of initially stable patients at risk for decompensation, and could be applied continuously for real-time estimates of near-term clinical deterioration.

ED patients are unique in the extent to which their underlying diagnoses and severity of illness are often unknown on initial presentation. While several prior studies have applied machine learning to the prediction of clinical events^18,32,40–47^, almost all have focused on general hospital ward, ICU, or operating room settings in which the patient has already undergone substantial evaluation. The few studies involving ED patients have seldom forecasted outcomes occurring within the ED visit itself^48,49^. Though high-resolution physiologic monitors are ubiquitous in the ED setting, few institutions retain the data they record. As the costs of storing and processing such data continue to fall, we anticipate that clinical prediction using real-time physiologic data will become increasingly routine.

Tree ensemble models often outperform deep learning models on structured data^50^, particularly on smaller datasets^51^. We adopted a hybrid modeling approach, using a gradient-boosted decision tree ensemble as our high-level modeling framework, and incorporating both engineered waveform features and deep learning-derived waveform embeddings as inputs to these models.

Unsurprisingly, trends in vital signs during post-triage monitoring are major predictors of subsequent abnormalities in the same vital sign. In predicting tachycardia or hypotension in the 90-minute window following assessment, most of the improvement in prediction accuracy (over baseline models using triage information alone) was achieved by modeling vital sign trends during the assessment period. In many cases, engineered or learned waveform features may be substantially correlated with vital signs (e.g., PAT with BP, HRV with HR, perfusion index with SpO2). In predicting hypoxia, however, the addition of the perfusion index nevertheless yielded a performance improvement over otherwise identical models without this information. For the prediction of tachycardia, second-order features appeared to be more useful for longer-range (120 min. window) compared to shorter-range (90 min. window) predictions, which may reflect diminishing prognostic value of simple vital sign trends at this longer horizon.

For prediction of hypotension and hypoxia, engineered waveform features including HRV measures and the PPG perfusion index were highlighted by SHAP analysis. The best-performing model for hypoxia, for example, uses SpO2 during the assessment period, as well as the perfusion index (derived from the PPG waveform) and HRV (measures derived from the ECG waveform) to reduce false positives compared to the baseline model. Though the best-performing model for hypotension prediction contains ECG/PPG waveform-derived embeddings, SHAP analysis does not heavily weight these features. This discrepancy may derive from correlations among embedding dimensions and other features. In the fully featured hypotension model, which performs similarly to the more restrictive, best-performing model, blood pressure trends are supplemented by perfusion index and an HRV metric, which has previously been associated with incipient hemodynamic collapse^52,53^. Overall, our feature analysis suggests that the assessment period is highly valuable for the prediction of hypotension, as the best-performing model is able to correctly reclassify visits that would otherwise be erroneously flagged as high-risk by a simpler model solely relying on lower blood pressures at triage.

Predicting new hypoxia was the most difficult task overall, and the best-performing model made broad use of available features. Indeed, the perfusion index, a measure of peripheral perfusion derived from the PPG waveform, was the single highest-weighted feature in the prediction of incipient hypoxia in initially normoxic patients. Previous work has not established a clear role for the perfusion index in predicting hypoxia^54^. Given that the perfusion index reflects the quality of the pulse oximetry signal^55^, we speculate that the addition of this feature may help the model calibrate the influence of SpO2 measurements and trends on predictions of subsequent hypoxia.

Our study has several limitations. Though we tested models on a chronologically later corpus of visits to simulate prospective validation, we had access to data from a single academic center, which may not generalize to other settings. Our outcome of interest, near-term decompensation of initially stable patients, represents a small but important proportion of all ED visits, and larger models are likely to benefit from a larger and more diverse number of training cases. Hypotension-prediction models exhibited the worst calibration to the underlying event distribution, which may result from the comparatively lower incidence of hypotension compared to the other abnormalities. Isotonic regression improved calibration, particularly for hypotension and hypoxia. We anticipate that training the models on larger datasets with more decompensation events will lead to further improvements in calibration, which will be essential to minimize distracting false alarms in clinical deployment. We used only the first 15 min of monitoring to simulate the benefit of a “secondary triage” model for subsequent decompensation, and because patients in our dataset are most reliably monitored early in the visit. Future research can extend this approach to a rolling prediction window, potentially including iterative model personalization based on the accuracy of earlier predictions. ECG and PPG waveforms captured in ED settings can be noisy due to frequent patient movement and transfers. While we preprocessed our waveform segments to filter out noisy segments, this filtering limits the length and number of usable waveforms. Given the limited size of our dataset and the predictive impact of well-described waveform features like HRV and PAT, we cannot claim to have learned all relevant latent features of the ECG and PPG waveforms. Finally, though we could identify the applications in which waveform embeddings contributed to correct predictions, we were limited in our ability to discern specific waveform features related to these predictions.

The decompensation of initially stable patients may be substantially predictable, using data already routinely collected in acute care settings. If prospectively validated, we propose that our prediction framework could be implemented in two complementary ways. First, as a supplement or modification to existing triage practices, a brief period of continuous monitoring could be performed in the waiting room, upon initial rooming, or even prior to hospital arrival via remote patient monitoring. Features would be automatically computed, and a risk score generated that could aid triage staff in prioritizing care for higher-risk patients. Prediction of specific physiologic abnormalities, rather than more commonly predicted aggregate or composite outcomes, can direct scarce clinician attention to a specific patient’s most time-sensitive diagnostic tests and modifiable risks. For instance, an initially normotensive patient at high risk for hypotension can be rapidly phenotyped for correctible derangements by bedside ultrasound^56^, while a normoxic patient at risk for hypoxia can receive noninvasive oxygen or ventilatory support, or evaluation by a respiratory therapist, prior to a decompensation requiring emergent ventilatory measures. Because our models rely on information collected passively by ubiquitous ECG and PPG sensors, these data could be obtained from standard monitors or from wearable devices in the waiting room or even in ambulatory settings. Second, our framework could be applied continuously to monitored patients, or when queried by a clinician, to provide a real-time estimate of a patient’s risk of near-term decompensation, thereby guiding management and disposition. Such an approach would make fuller use of continuously collected waveforms and could also incorporate data on physiologic responses to medication administration and other interventions.

## Methods

### Data sources and transformations

We studied 19,847 adult visits to monitored beds of the Stanford Health Care Emergency Department that occurred between August 1st 2020 and April 30th 2022. For each visit, we observed patient age, self-reported gender, and vital signs at triage: heart rate (HR), systolic (SBP) and diastolic (DBP) blood pressure, mean arterial blood pressure (MAP = 1/3 SBP + 2/3 DBP), oxygen saturation (SpO2), temperature, and respiratory rate (RR). We obtained vital signs (HR, RR, SpO2, MAP) and continuous lead II ECG and PPG waveforms through the entire ED visit from Philips IntelliVue bedside monitors. We obtained intermittent temperature measurements from nursing charts. We used the one-minute means of HR, RR, and SpO2 measurements to reduce impact of localized variation or noise. We calculated the Modified Early Warning Score (MEWS) at each minute^37^, omitting neurologic status (which was not consistently documented in our data), and carrying forward intermittently observed vital signs (i.e., blood pressure and temperature). In order to predict new or unexpected decompensation, we included only visits with grossly normal vital signs at triage and during the first 15 min of monitoring (HR ≤ 110, SpO2 ≥ 90, MAP ≥ 65), and excluded visits without at least one measurement of each vital sign and waveform (Supplementary Figure 2).

For each ED visit, we defined the assessment period as the first 15 min of monitoring after the patient was roomed. We used vital signs and ECG/PPG waveforms from the assessment period, in addition to patient age, gender, triage vital signs, and chief complaint, to predict subsequent physiologic decompensation: tachycardia (HR > 110), hypoxia (SpO2 < 90), or hypotension (MAP < 65) within 90 min after the assessment period. In supplemental analyses, we assessed a previously validated composite outcome of vital sign derangements (MEWS ≥ 4), and 60- and 120-minute prediction windows for all outcomes.

We developed separate models for each abnormality (tachycardia, hypoxia, hypotension, and MEWS ≥ 4) on the same cohort of initially stable patients. We divided the cohort into training (75%), validation (12.5%), and hold-out test sets (12.5%), with the test set containing visits occurring after those in the training and validation sets, so as to simulate prospective validation. The training and validation sets contained data from approximately the first 18 months of data collection, and the test sets contained visits from the last 3 months of data collection. We used scikit-learn’s ‘GroupShuffleSplit’ package for the grouped splitting based on patient identification, such that the train, validation, and hold-out test sets had no patient overlap (in the case of patients with multiple visits)^57^.

### Features used for prediction of decompensation

We combined four classes of features in the prediction of clinical deterioration: features observed at ED triage, features recorded directly by bedside monitors during the assessment period, features engineered from ECG and PPG waveforms, and deep learning-derived embedding representations of ECG and PPG waveforms. Triage features included patient age, gender, Emergency Severity Index (ESI) assigned at triage, vital signs at triage (HR, RR, SpO2, MAP, SBP, DBP), and 46 indicator variables for categories of chief complaint at triage. Directly monitored features included first vital signs during the assessment period (HR, RR, SpO2, MAP), and the coefficients of linear trends in these features during the assessment period. Features engineered from ECG and PPG waveforms included several measures of heart rate variability (HRV) derived from beat-to-beat RR interval and from the ECG waveform itself, and pulse arrival time (PAT) as measured from concurrent ECG and PPG waveforms. Finally, we used transformers to generate waveform embeddings from 60-second ECG and PPG samples.

### Waveform data and preprocessing

Continuous ECG and PPG waveforms are subject to artifacts and gaps in recording due to sensor detachment and patient movement. We developed a pre-processing strategy to select the first 60-second window of the assessment period in which both ECG and PPG waveforms demonstrated acceptable quality. For ECG waveforms, we used Hamilton’s method^58^ to identify R-peaks and determine heart rate, and discarded waveforms without a detectable heart rate between 25–300 beats per minute, or with outlier amplitudes exceeding 4 mV. For PPG waveforms, we measured skewness, matching of systolic waves, and presence of stationary segments, using signal quality thresholds based on prior studies^59,60^. We discarded visits without acceptable ECG and PPG waveforms in the same 60-second window. To reduce noise, we applied a 3–45 Hz bandpass filter to the ECG waveforms and a 4th-order Butterworth filter to the PPG waveforms. ECG waveforms were downsampled from 500 Hz to 125 Hz to match PPG waveform frequency.

### Measures of heart rate variability

Prior research has established time- and frequency-domain measures of heart rate variability (HRV) for clinical prediction tasks^61^. We used beat-to-beat RR intervals from continuous lead II ECG to generate the following time-domain HRV measures: standard deviation of the RR intervals (SDRR), percentage of successive RR intervals that differ by more than 50 ms (pRR50), root mean square of successive RR interval differences (RMSSD), the HRV triangular index as calculated by an approximation of the integral of the density of the RR interval histogram divided by its height, and the width of the RR interval histogram (TINN). We produced a frequency-domain representation of 60 s ECG waveforms by applying a Fourier transform to estimate the power spectral density of the ECG signal. We then calculated the following frequency-domain HRV measures: peak frequency of the low-frequency band (0.04–0.15 Hz), peak frequency of the high-frequency band (0.15–0.4 Hz), absolute power of the low-frequency band, absolute power of the high-frequency band, relative power of the low-frequency band, relative power of the high-frequency band, and the ratio of low-frequency to high-frequency power^61^.

### Pulse arrival time

For each 60-second segment of aligned ECG and PPG waveforms, we calculated the pulse arrival time (PAT): the mean time between peaks of the ECG and PPG signal, representing the delay between electrical systole in the heart and resulting peripheral blood flow. We found waveform peaks using scipy’s ‘find_peaks’ function^57^, discarded ECG-PPG peak pairs appearing to be further apart than the corresponding ECG RR interval, then measured the mean delay between valid pairs of ECG and PPG peaks.

### Deep learning representations of ECG/PPG waveforms

To extract additional features from ECG and PPG waveforms, we modified a transformer-based deep neural network initially developed for the classification of static 12-lead ECGs^62^. The model consists of a series of 1D convolutional layers to extract waveform features, followed by transformer blocks and fully connected layers to represent waveform features relevant to the prediction task. We initialized the model with weights from its original application for the detection of ECG rhythm abnormalities^62^, reasoning that these pre-trained weights would extract some general waveform features relevant to any downstream task. We adapted Natarajan et al.’s model to predict ED decompensation by adding input channels to the convolutional layers for both ECG and PPG waveforms, and to the final fully connected layers to produce a lower-dimensional embedding layer whose values could be used as inputs to our final models for the prediction of decompensation. Figure 1 depicts our overall data sampling and modeling strategy.

We trained each deep model for 60 epochs, using a cyclic learning rate scheduler and applying a binary cross entropy loss against labels of new tachycardia, hypoxia, or hypotension. The prediction performance of these deep models alone after supervised training when evaluated on the test set is reported in Supplementary Table 16. We tested various embedding lengths for waveforms as inputs to light gradient boosting machine (LGBM) models, in which we combined waveform embeddings with other features for the prediction of physiologic decompensation (Supplementary Tables 17-18). We selected 4-dimensional embeddings for each of the PPG and ECG waveform inputs.

### Gradient-boosted decision tree classifiers

We combined the features above in light gradient boosting machine (LGBM) classifiers^63^, a decision tree ensemble model that has proven effective in clinical classification tasks^64^. We trained separate LGBMs for each prediction task and set of input features. We fine-tuned each model’s hyperparameters using the Python package ‘verstack’^65^ for 100 trials, optimizing for AUROC on the validation set.

We retrained 100 LGBM models (with early_stopping_rounds set to 50) for each task and each set of input features by varying the models’ ‘random_state’ hyperparameter and chose the best-performing model based on AUROC on the validation cohort to then evaluate on the test set. Given the inherent stochasticity of the underlying model architecture, this approach enables us to select a model that more likely represents the peak performance by treating the model’s initial random state as an additional hyperparameter to optimize.

### Statistical analysis

We performed statistical analysis of model performance via bootstrapping to account for uncertainty derived from randomness in the test set patient data, and without imposing distributional assumptions. For each model, we computed 95% CIs for AUROC and AUPRC using bootstrap resampling with 10,000 replicates. All descriptive statistical tests (e.g., t-tests) are two-sided.

### Model interpretability

We evaluated feature importance using SHapley Additive exPlanations (SHAP), which uses a game-theoretic mechanism to assign a contribution score to each feature^66^. We calculated the mean absolute SHAP value for each feature across each example in the test set and divided by the sum of SHAP values across all features to produce a score for each feature’s overall contribution to model predictions of clinical decompensation. To determine directionality of contributions (i.e., whether greater values of a feature contributed towards a positive or negative prediction), we calculated Pearson’s correlation coefficient between a feature’s value and its SHAP score.

### Analysis of correctly reclassified cases

To determine features of visits correctly classified by our best-performing models but incorrectly classified using the baseline triage model, we set operating points for each model to 85% classification sensitivity on the validation set. We then identified the visits correctly classified by the best-performing model for each task, and incorrectly classified by the baseline triage model. We compared features of these visits to visits not reclassified by the best-performing models using two-sided t-tests.

### Model calibration plots

We produced calibration plots for baseline and best-performing models for each task, in the 90-minute prediction window. We ranked visits by predicted probability of decompensation, divided predictions into quintiles, and for each quintile of predicted decompensation calculated mean predicted probability and proportion of true positives. We applied isotonic regression, fit on the validation set, to the best-performing models.

### Alignment between individual decompensation predictions and MEWS

For each individual abnormality predicted (tachycardia, hypotension, hypoxia), we produced dichotomous predictions of decompensation using operating points selected for 85% validation sensitivity. For visits predicted positive or negative for decompensation in the test set, we recorded the number of patients reaching MEWS ≥ 4 during the prediction window. “Alignment” between predicted decompensation and MEWS is the proportion of patients reaching MEWS ≥ 4 who were predicted to decompensate, or the proportion of patients with maximum MEWS < 4 predicted not to decompensate.

### Software

Continuous monitor data was extracted from the Stanford Health Care Philips Data Warehouse using Philips PIC iX DWC Toolkit (C.03.31). All analyses were performed using Python (3.9.7). Data processing was performed using numpy (1.21.6), pandas (1.4.2), h5py (3.6.0) and scikit-learn (1.0.1). Cohort statistical analysis was performed using scipy (1.8.0). HRV/PTT feature extraction was performed using scipy (see above) and matplotlib (3.5.1). Transformer training and evaluation was performed using torch (1.10.2 + cu111), pytorch_lightning (1.6.1), torchmetrics (0.8.0), edm (0.0.4) and wandb (0.12.14). Additionally, the edm package uses biosppy (0.6.1) and vital-sqi (0.1.0). LGBM model training, tuning and evaluation was performed using lightgbm (3.3.0), scikit-learn (see above) and verstack (3.2.3). Secondary analyses of model performance were performed using shap (0.40.0), scikit-learn (see above), matplotlib (see above) and scipy (see above).

### Ethics

The study was approved by the Institutional Review Board of Stanford University, with a waiver of consent for retrospective research on anonymized data.

### Reporting summary

Further information on research design is available in the Nature Research Reporting Summary linked to this article.

## Supplementary information

Supplemental Information

REPORTING SUMMARY

## Data Availability

A de-identified dataset sufficient to reproduce main results is available from the corresponding author upon reasonable request. The original study dataset contains protected health information and cannot be distributed.

## Code availability

The code used for data pre-processing, model training, performance evaluation, and data analysis is available in the following repositories: https://github.com/rajpurkarlab/ed-monitor-decompensation. https://github.com/stanfordmlgroup/ed-monitor-data.
