# Supplementary material for: Predicting patient decompensation from continuous physiologic monitoring in the emergency department
Source: NPJ Digit Med. 2023 Apr 4;6:60. doi: 10.1038/s41746-023-00803-0 (PMC10073111; doi:10.1038/s41746-023-00803-0)
Supplement: Supplementary file 1 — Supplemental Information [file 41746_2023_803_MOESM1_ESM.pdf]

Supplementary Information: Predicting Patient Decompensation from Continuous Physiologic Monitoring in the Emergency Department

Supplementary Table 1. Effect of feature types on AUROC for prediction of tachycardia, hypotension, and hypoxia (90min window)....... 2

Supplementary Table 2. Effect of feature types on AUPRC for prediction of tachycardia, hypotension, and hypoxia (90min window). 3

Supplementary Table 3. Test characteristics for baseline and best-performing models at three operating points (90min window). 4

Supplementary Table 4. Confusion matrices of triage-only and best-performing models on the test set (90min window).......................... 5

Supplementary Table 5. Characteristics of patients for each decompensation cohort (60min window). 6

Supplementary Table 6. Effect of feature types on AUROC for prediction of tachycardia, hypotension, and hypoxia (60min window)....... 7

Supplementary Table 7. Effect of feature types on AUPRC for prediction of tachycardia, hypotension, and hypoxia (60min window). 8

Supplementary Table 8. Characteristics of patients for each decompensation cohort (120min window). 9

Supplementary Table 9. Effect of feature types on AUROC for prediction of tachycardia, hypotension, and hypoxia (120min window)... 10

Supplementary Table 10. Effect of feature types on AUPRC for prediction of tachycardia, hypotension, and hypoxia (120min window). 11

Supplementary Table 11. Effect of feature types on AUROC for prediction of MEWS ≥ 4 (all prediction windows). 12

Supplementary Table 12. Effect of feature types on AUPRC for prediction of MEWS ≥ 4 (all prediction windows). 13

Supplementary Table 13. Alignment between decompensation predictions and maximum MEWS≥4 in the test set (90min window). 14

Supplementary Figure 1. Calibration plots for triage-only baseline and best-performing models for each outcome (90min window)........ 15

Supplementary Table 14. Contribution of feature types to model predictions (90min window). 16

Supplementary Table 15. Differences in feature values among correctly reclassified visits (90min window). 17

Supplementary Figure 2. Cohort creation. 20

Supplementary Table 16. Predictive performance of waveform transformer alone (90min window). 21

Supplementary Table 17. Effect of waveform embedding length on AUROC for prediction of decompensation (90min window). 22

Supplementary Table 18. Effect of waveform embedding length on AUPRC for prediction of decompensation (90min window). 23

Supplementary Table 1. Effect of feature types on AUROC for prediction of tachycardia, hypotension, and hypoxia (90min window).

AUROC values and bootstrapped 95% CIs represent performance on the test set. Each row represents a separately tuned and trained model. “Triage” variables include age, gender, triage vital signs, and chief complaint. “VS trend” denotes first vital signs from continuous monitoring, and the linear trends of each vital sign over a 15-minute assessment period. “PAT” denotes pulse arrival time, calculated from the ECG and PPG waveforms. “HRV” is a suite of heart rate variability measures. “Perfusion” is the perfusion index. “Waveform” indicates an 8-dimensional embedding generated from a transformer model, with 4 features each from the PPG and ECG waveforms. The best-performing model for each outcome is bolded.

Triage	VS trend	PAT	HRV	Perfusion	Waveform	Tachycardia		Hypotension		Hypoxia	
						AUROC (95% CI)	AUROC difference from triage-only model (95% CI)	AUROC (95% CI)	AUROC difference from triage-only model (95% CI)	AUROC (95% CI)	AUROC difference from triage-only model (95% CI)
■	■				■	0.826 (0.788 - 0.861)	0.025 (-0.006 - 0.058)	**0.802 (0.747 - 0.856)**	**0.073 (0.034 - 0.112)**	0.651 (0.615 - 0.685)	0.048 (0.014 - 0.083)
■	■	■	■	■	■	0.824 (0.785 - 0.859)	0.023 (-0.008 - 0.056)	0.792 (0.729 - 0.851)	0.063 (0.013 - 0.111)	0.711 (0.679 - 0.743)	0.109 (0.070 - 0.148)
■	■	■	■	■		0.834 (0.798 - 0.869)	0.034 (0.003 - 0.066)	0.764 (0.698 - 0.827)	0.035 (-0.012 - 0.078)	**0.713 (0.680 - 0.745)**	**0.111 (0.074 - 0.147)**
■	■	■	■			0.834 (0.797 - 0.869)	0.034 (0.001 - 0.069)	0.767 (0.702 - 0.830)	0.039 (-0.010 - 0.086)	0.646 (0.610 - 0.681)	0.044 (0.009 - 0.078)
■	■		■			0.836 (0.799 - 0.870)	0.036 (0.006 - 0.067)	0.782 (0.719 - 0.840)	0.053 (0.004 - 0.099)	0.634 (0.597 - 0.671)	0.032 (-0.001 - 0.065)
■	■	■				0.826 (0.788 - 0.861)	0.025 (-0.004 - 0.057)	0.791 (0.735 - 0.847)	0.062 (0.021 - 0.102)	0.641 (0.604 - 0.677)	0.038 (0.005 - 0.070)
■	■					**0.836 (0.800 - 0.870)**	**0.036 (0.003 - 0.070)**	0.782 (0.726 - 0.838)	0.053 (0.013 - 0.093)	0.645 (0.609 - 0.681)	0.043 (0.009 - 0.076)
■						0.800 (0.756 - 0.842)		0.729 (0.666 - 0.791)		0.602 (0.565 - 0.640)	

Supplementary Table 2. Effect of feature types on AUPRC for prediction of tachycardia, hypotension, and hypoxia (90min window).

Area under the precision-recall curve (AUPRC) is evaluated on the test set. Each row represents a separately tuned and trained model. “Triage” variables include age, gender, triage vital signs, and chief complaint. “VS trend” denotes first vital signs from continuous monitoring, and the linear trends of each vital sign over a 15-minute assessment period. “PAT” denotes pulse arrival time, calculated from the ECG and PPG waveforms. “HRV” is a suite of heart rate variability measures. “Perfusion” is the perfusion index. “Waveform” indicates an 8-dimensional embedding generated from a transformer model, with 4 features each from the PPG and ECG waveforms.

Triage	VS Trend	PAT	HRV	Perfusion	Waveform	Tachycardia AUPRC	Hypotension AUPRC	Hypoxia AUPRC
■	■				■	0.238 (0.178 - 0.318)	0.180 (0.103 - 0.282)	0.206 (0.170 - 0.256)
■	■	■	■	■	■	0.232 (0.176 - 0.312)	0.177 (0.100 - 0.275)	0.238 (0.198 - 0.290)
■	■	■	■	■		0.254 (0.194 - 0.337)	0.159 (0.091 - 0.268)	0.244 (0.203 - 0.298)
■	■	■	■			0.240 (0.183 - 0.317)	0.161 (0.089 - 0.253)	0.225 (0.183 - 0.277)
■	■		■			0.240 (0.183 - 0.321)	0.163 (0.091 - 0.257)	0.219 (0.178 - 0.271)
■	■	■				0.218 (0.168 - 0.292)	0.167 (0.094 - 0.264)	0.207 (0.170 - 0.257)
■	■					0.244 (0.188 - 0.328)	0.173 (0.096 - 0.271)	0.207 (0.170 - 0.257)
■						0.246 (0.182 - 0.323)	0.109 (0.060 - 0.199)	0.166 (0.139 - 0.205)

Supplementary Table 3. Test characteristics for baseline and best-performing models at three operating points (90min window).

We set operating points for validation sensitivity of 0.85, 0.95 and 0.99 and computed test sensitivity, specificity, negative predictive value (NPV) and positive predictive value (PPV) for both the triage-only and best-performing models. We report the test characteristics and bootstrapped 95% CIs.

Tachycardia

	**Triage features only**			**Best-performing model**		
Validation sensitivity	0.85	0.95	0.99	0.85	0.95	0.99
Test sensitivity (95% CI)	0.817 (0.746 - 0.882)	0.929 (0.880 - 0.97)	0.968 (0.934 - 0.993)	0.786 (0.711 - 0.857)	0.929 (0.880 - 0.970)	1.00 (1.00 - 1.00)
Test specificity (95% CI)	0.608 (0.588 - 0.627)	0.286 (0.268 - 0.304)	0.141 (0.127 - 0.155)	0.740 (0.723 - 0.758)	0.443 (0.423 - 0.464)	0.074 (0.064 - 0.085)
NPV (95% CI)	0.984 (0.977 - 0.990)	0.987 (0.977 - 0.994)	0.988 (0.975 - 0.997)	0.985 (0.979 - 0.990)	0.991 (0.985 - 0.996)	1.00 (1.00 - 1.00)
PPV (95% CI)	0.101 (0.083 - 0.120)	0.065 (0.054 - 0.077)	0.057 (0.048 - 0.067)	0.140 (0.115 - 0.166)	0.082 (0.069 - 0.097)	0.055 (0.046 - 0.065)

Hypotension

	**Triage features only**			**Best-performing model**		
Validation sensitivity	0.85	0.95	0.99	0.85	0.95	0.99
Test sensitivity (95% CI)	0.661 (0.543 - 0.778)	0.887 (0.800 - 0.962)	1.00 (1.00 - 1.00)	0.742 (0.633 - 0.848)	0.839 (0.742 - 0.925)	1.00 (1.00 - 1.00)
Test specificity (95% CI)	0.668 (0.649 - 0.687)	0.358 (0.339 - 0.377)	0.042 (0.034 - 0.050)	0.690 (0.671 - 0.708)	0.578 (0.559 - 0.598)	0.168 (0.153 - 0.184)
NPV (95% CI)	0.987 (0.981 - 0.992)	0.992 (0.985 - 0.998)	1.00 (1.00 - 1.00)	0.990 (0.986 - 0.995)	0.993 (0.988 - 0.997)	1.00 (1.00 - 1.00)
PPV (95% CI)	0.049 (0.035 - 0.064)	0.034 (0.026 - 0.043)	0.026 (0.020 - 0.033)	0.058 (0.043 - 0.074)	0.049 (0.036 - 0.062)	0.030 (0.023 - 0.038)

Hypoxia

	**Triage features only**			**Best-performing model**		
Validation sensitivity	0.85	0.95	0.99	0.85	0.95	0.99
Test sensitivity (95% CI)	0.847 (0.802 - 0.889)	0.910 (0.874 - 0.943)	0.974 (0.953 - 0.992)	0.869 (0.827 - 0.908)	0.970 (0.949 - 0.989)	0.985 (0.969 - 0.996)
Test specificity (95% CI)	0.239 (0.221 - 0.257)	0.118 (0.104 - 0.131)	0.043 (0.034 - 0.051)	0.365 (0.344 - 0.385)	0.189 (0.173 - 0.205)	0.094 (0.082 - 0.106)
NPV (95% CI)	0.928 (0.905 - 0.948)	0.915 (0.881 - 0.945)	0.931 (0.877 - 0.978)	0.958 (0.944 - 0.971)	0.981 (0.967 - 0.993)	0.981 (0.960 - 0.996)
PPV (95% CI)	0.119 (0.105 - 0.134)	0.112 (0.099 - 0.125)	0.110 (0.098 - 0.123)	0.143 (0.126 - 0.160)	0.127 (0.113 - 0.142)	0.117 (0.104 - 0.131)

Supplementary Table 4. Confusion matrices of triage-only and best-performing models on the test set (90min window).

Operating point selected for validation sensitivity of 0.85.

Tachycardia

	**Triage features only**		**Best-performing model**	
	Actual positive	Actual Negative	Actual positive	Actual Negative
Predicted positive	103	918	99	608
Predicted negative	23	1422	27	1732

Hypotension

	**Triage features only**		**Best-performing model**	
	Actual positive	Actual Negative	Actual positive	Actual Negative
Predicted positive	41	798	46	746
Predicted negative	21	1606	16	1658

Hypoxia

	**Triage features only**		**Best-performing model**	
	Actual positive	Actual Negative	Actual positive	Actual Negative
Predicted positive	227	1673	233	1396
Predicted negative	41	525	35	802

Supplementary Table 5. Characteristics of patients for each decompensation cohort (60min window).

Differences were evaluated with Wilcoxon rank-sum tests for numeric variables, and chi-squared tests for categorical variables.

	Tachycardia			Hypoxia			Hypotension		
**Characteristic**	**Decomp. (n=922)**	**No Decomp. (n=17803)**	**p-value of diff.**	**Decomp. (n=1568)**	**No Decomp. (n=17157)**	**p-value**	**Decomp. (n=363)**	**No Decomp. (n=18362)**	**p-value of diff.**
Age in years, median [IQR]	54.0 [36.0-71.0]	61.0 [44.0-75.0]	< 0.001	65.0 [50.0-79.0]	60.0 [43.0-75.0]	< 0.001	64.0 [42.0-79.0]	61.0 [43.0-75.0]	0.18
Gender, n (%)									
Female	483 (52.39)	8981 (50.46)		794 (50.64)	8670 (50.55)		208 (57.3)	9256 (50.42)	
Male	439 (47.61)	8817 (49.54)	0.254	774 (49.36)	8482 (49.45)	0.946	155 (42.7)	9101 (49.58)	0.009
Triage Vital Signs, median [IQR]									
SpO2, %	99.0 [97.0-100.0]	99.0 [97.0-100.0]	0.167	98.0 [96.0-100.0]	99.0 [98.0-100.0]	< 0.001	99.0 [97.0-100.0]	99.0 [97.0-100.0]	0.013
Respiratory Rate	18.0 [16.0-20.0]	18.0 [16.0-19.0]	< 0.001	18.0 [16.0-20.0]	18.0 [16.0-19.0]	< 0.001	18.0 [16.0-20.0]	18.0 [16.0-19.0]	0.526
Heart Rate	97.0 [88.0-103.0]	81.0 [72.0-91.0]	< 0.001	83.0 [72.0-94.0]	82.0 [72.0-92.0]	0.052	81.0 [70.0-93.0]	82.0 [72.0-92.0]	0.321
Systolic BP	137.0 [124.0-153.0]	138.0 [123.0-153.0]	0.891	135.0 [120.0-152.0]	138.0 [123.0-153.0]	< 0.001	116.0 [104.5-131.0]	138.0 [123.0-153.0]	< 0.001
Diastolic BP	83.5 [73.0-94.0]	79.0 [69.0-89.0]	< 0.001	79.0 [68.0-90.0]	79.0 [69.0-89.0]	0.507	66.0 [57.5-79.0]	79.0 [69.0-89.0]	< 0.001
Mean Arterial BP	101.7 [91.3-112.3]	98.7 [88.7-109.0]	< 0.001	98.0 [86.7-109.0]	99.0 [89.0-109.3]	0.015	81.7 [74.7-96.3]	99.0 [89.0-109.3]	< 0.001
ED Visit Length, hours [IQR]	5.96 [4.39-8.13]	5.63 [4.12-7.57]	< 0.001	6.1 [4.58-8.11]	5.6 [4.08-7.55]	< 0.001	5.92 [4.39-8.14]	5.65 [4.12-7.58]	0.028
Emergency Severity Index, n (%)									
Level 1	15 (1.63)	144 (0.81)	0.008	20 (1.28)	139 (0.81)	0.055	15 (4.13)	144 (0.78)	< 0.001
Level 2	335 (36.33)	5354 (30.07)	< 0.001	534 (34.06)	5155 (30.05)	< 0.001	143 (39.39)	5546 (30.20)	< 0.001
Level 3	544 (59.00)	11923 (66.97)	< 0.001	975 (62.18)	11492 (66.98)	< 0.001	199 (54.82)	12268 (66.81)	< 0.001
Level 4	21 (2.28)	277 (1.56)	0.088	26 (1.66)	272 (1.59)	0.825	4 (1.10)	294 (1.60)	0.452
Level 5	1 (0.11)	11 (0.06)	0.585	4 (0.26)	8 (0.05)	0.002	0 (0.0)	12 (0.07)	0.626
Admitted, n (%)	448 (48.59)	7179 (40.32)	< 0.001	792 (50.51)	6835 (39.84)	< 0.001	207 (57.02)	7420 (40.41)	< 0.001

Supplementary Table 6. Effect of feature types on AUROC for prediction of tachycardia, hypotension, and hypoxia (60min window).

AUROC values and bootstrapped 95% CIs represent performance on the test set. Each row represents a separately tuned and trained model. “Triage” variables include age, gender, triage vital signs, and chief complaint. “VS trend” denotes first vital signs from continuous monitoring, and the linear trends of each vital sign over a 15-minute assessment period. “PAT” denotes pulse arrival time, calculated from the ECG and PPG waveforms. “HRV” is a suite of heart rate variability measures. “Perfusion” is the perfusion index. “Waveform” indicates an 8-dimensional embedding generated from a transformer model, with 4 features each from the PPG and ECG waveforms. The best-performing model for each outcome is bolded.

Triage	VS trend	PAT	HRV	Perfusion	Waveform	Tachycardia		Hypotension		Hypoxia	
						AUROC (95% CI)	AUROC difference from triage-only model (95% CI)	AUROC (95% CI)	AUROC difference from triage-only model (95% CI)	AUROC (95% CI)	AUROC difference from triage-only model (95% CI)
■	■				■	0.823 (0.779 - 0.864)	0.017 (-0.013 - 0.049)	0.754 (0.673 - 0.829)	0.027 (-0.029 - 0.087)	0.631 (0.589 - 0.674)	0.003 (-0.035 - 0.043)
■	■	■	■	■	■	0.815 (0.770 - 0.855)	0.008 (-0.03 - 0.049)	0.756 (0.678 - 0.829)	0.029 (-0.026 - 0.087)	**0.712 (0.673 - 0.75)**	**0.085 (0.041 - 0.129)**
■	■	■	■	■		0.822 (0.779 - 0.861)	0.016 (-0.023 - 0.056)	**0.764 (0.683 - 0.838)**	**0.037 (-0.023 - 0.100)**	0.702 (0.661 - 0.742)	0.075 (0.029 - 0.120)
■	■	■	■			0.821 (0.78 - 0.859)	0.015 (-0.023 - 0.055)	0.762 (0.684 - 0.835)	0.035 (-0.021 - 0.095)	0.66 (0.618 - 0.701)	0.032 (-0.014 - 0.078)
■	■		■			0.823 (0.781 - 0.862)	0.017 (-0.02 - 0.056)	0.743 (0.658 - 0.823)	0.016 (-0.047 - 0.08)	0.646 (0.605 - 0.688)	0.019 (-0.023 - 0.06)
■	■	■				**0.824 (0.781 - 0.863)**	**0.018 (-0.018 - 0.056)**	0.764 (0.686 - 0.834)	0.037 (-0.012 - 0.088)	0.637 (0.596 - 0.677)	0.009 (-0.033 - 0.052)
■	■					0.818 (0.773 - 0.859)	0.012 (-0.023 - 0.047)	0.76 (0.682 - 0.832)	0.033 (-0.021 - 0.09)	0.635 (0.593 - 0.677)	0.007 (-0.037 - 0.052)
■						0.806 (0.758 - 0.852)		0.727 (0.645 - 0.802)		0.628 (0.586 - 0.669)	

Supplementary Table 7. Effect of feature types on AUPRC for prediction of tachycardia, hypotension, and hypoxia (60min window).

Area under the precision-recall curve (AUPRC) is evaluated on the test set. Each row represents a separately tuned and trained model. “Triage” variables include age, gender, triage vital signs, and chief complaint. “VS trend” denotes first vital signs from continuous monitoring, and the linear trends of each vital sign over a 15-minute assessment period. “PAT” denotes pulse arrival time, calculated from the ECG and PPG waveforms. “HRV” is a suite of heart rate variability measures. “Perfusion” is the perfusion index. “Waveform” indicates an 8-dimensional embedding generated from a transformer model, with 4 features each from the PPG and ECG waveforms.

Triage	VS Trend	PAT	HRV	Perfusion	Waveform	Tachycardia AUPRC	Hypotension AUPRC	Hypoxia AUPRC
■	■				■	0.164 (0.121 - 0.231)	0.181 (0.089 - 0.297)	0.147 (0.113 - 0.199)
■	■	■	■	■	■	0.151 (0.113 - 0.213)	0.144 (0.07 - 0.254)	0.189 (0.146 - 0.244)
■	■	■	■	■		0.154 (0.115 - 0.215)	0.167 (0.082 - 0.274)	0.182 (0.141 - 0.234)
■	■	■	■			0.152 (0.113 - 0.216)	0.158 (0.079 - 0.273)	0.149 (0.117 - 0.198)
■	■		■			0.149 (0.113 - 0.207)	0.161 (0.076 - 0.268)	0.149 (0.115 - 0.201)
■	■	■				0.151 (0.113 - 0.203)	0.158 (0.078 - 0.264)	0.137 (0.106 - 0.184)
■	■					0.145 (0.109 - 0.195)	0.165 (0.080 - 0.278)	0.137 (0.107 - 0.184)
■						0.198 (0.138 - 0.279)	0.098 (0.048 - 0.179)	0.123 (0.098 - 0.162)

Supplementary Table 8. Characteristics of patients for each decompensation cohort (120min window).

Differences were evaluated with Wilcoxon rank-sum tests for numeric variables, and chi-squared tests for categorical variables.

Characteristic	Tachycardia			Hypoxia			Hypotension		
	**Decomp. (n=1474)**	**No Decomp. (n=19052)**	**p-value of diff.**	**Decomp. (n=2783)**	**No Decomp. (n=17743)**	**p-value of diff.**	**Decomp. (n=556)**	**No Decomp. (n=19970)**	**p-value of diff.**
Age in years, median [IQR]	54.0 [36.0-71.0]	61.0 [44.0-75.0]	< 0.001	65.0 [49.0-79.0]	60.0 [42.0-74.0]	< 0.001	63.5 [41.0-78.0]	61.0 [43.0-75.0]	0.31
Female, n (%)	761 (51.63)	9771 (51.3)	0.808	1461 (52.5)	9071 (51.14)	0.183	335 (60.25)	10197 (51.07)	< 0.001
Male, n (%)	713 (48.37)	9276 (48.7)		1322 (47.5)	8667 (48.86)		221 (39.75)	9768 (48.93)	
Triage Vital Signs, median [IQR]									
SpO2, %	99.0 [97.0-100.0]	99.0 [97.0-100.0]	0.008	98.0 [97.0-100.0]	99.0 [98.0-100.0]	< 0.001	99.0 [97.0-100.0]	99.0 [97.0-100.0]	0.041
Respiratory Rate	18.0 [16.0-20.0]	18.0 [16.0-19.0]	< 0.001	18.0 [16.0-20.0]	18.0 [16.0-19.0]	< 0.001	18.0 [16.0-20.0]	18.0 [16.0-19.0]	0.56
Heart Rate	96.0 [87.0-103.0]	81.0 [72.0-91.0]	< 0.001	83.0 [73.0-93.0]	82.0 [72.0-92.0]	0.029	81.0 [70.0-92.0]	82.0 [72.0-92.0]	0.149
Systolic BP	136.0 [122.0-151.0]	138.0 [123.0-153.0]	0.076	135.0 [120.0-151.0]	138.0 [123.0-153.0]	< 0.001	118.0 [106.0-133.0]	138.0 [123.0-153.0]	< 0.001
Diastolic BP	82.0 [72.0-93.0]	79.0 [69.0-89.0]	< 0.001	78.0 [68.0-89.0]	79.0 [69.0-89.0]	0.049	67.0 [59.0-80.0]	79.0 [69.0-89.0]	< 0.001
Mean Arterial BP	100.7 [90.3-110.7]	98.7 [88.3-109.0]	< 0.001	97.7 [87.0-108.3]	99.0 [89.0-109.3]	< 0.001	84.0 [75.7-96.3]	99.0 [89.0-109.3]	< 0.001
ED Visit Length, hours [IQR]	6.07 [4.5-8.15]	5.65 [4.12-7.58]	< 0.001	6.25 [4.68-8.12]	5.58 [4.07-7.53]	< 0.001	6.01 [4.40-8.0075]	5.67 [4.13-7.6]	0.004
Emergency Severity Index, n (%)									
Level 1	17 (1.15)	143 (0.75)	0.09	22 (0.79)	138 (0.78)	0.943	17 (3.06)	143 (0.72)	< 0.001
Level 2	540 (36.64)	5543 (29.09)	< 0.001	896 (32.2)	5187 (29.23)	0.001	215 (38.67)	5868 (29.38)	< 0.001
Level 3	882 (59.84)	12963 (68.04)	< 0.001	1800 (64.68)	12045 (67.89)	< 0.001	316 (56.83)	13529 (67.75)	< 0.001
Level 4	25 (1.7)	294 (1.54)	0.647	44 (1.58)	275 (1.55)	0.902	6 (1.08)	313 (1.57)	0.359
Level 5	1 (0.07)	11 (0.06)	0.877	4 (0.14)	8 (0.05)	0.045	0 (0.0)	12 (0.06)	0.563
Admitted, n (%)	727 (49.32)	7575 (39.76)	< 0.001	1332 (47.86)	6970 (39.28)	< 0.001	295 (53.06)	8007 (40.1)	< 0.001

Supplementary Table 9. Effect of feature types on AUROC for prediction of tachycardia, hypotension, and hypoxia (120min window).

AUROC values and bootstrapped 95% CIs represent performance on the test set. Each row represents a separately tuned and trained model. “Triage” variables include age, gender, triage vital signs, and chief complaint. “VS trend” denotes first vital signs from continuous monitoring, and the linear trends of each vital sign over a 15-minute assessment period. “PAT” denotes pulse arrival time, calculated from the ECG and PPG waveforms. “HRV” is a suite of heart rate variability measures. “Perfusion” is the perfusion index. “Waveform” indicates an 8-dimensional embedding generated from a transformer model, with 4 features each from the PPG and ECG waveforms. The best-performing model for each outcome is bolded.

Triage	VS trend	PAT	HRV	Perfusion	Waveform	Tachycardia		Hypotension		Hypoxia	
						**AUROC (95% CI)**	**AUROC difference from triage-only model (95% CI)**	**AUROC (95% CI)**	**AUROC difference from triage-only model (95% CI)**	**AUROC (95% CI)**	**AUROC difference from triage-only model (95% CI)**
■	■				■	0.807 (0.771 - 0.841)	0.028 (-0.001 - 0.058)	0.775 (0.714 - 0.829)	0.033 (-0.015 - 0.079)	0.632 (0.599 - 0.663)	0.014 (-0.017 - 0.044)
■	■	■	■	■	■	**0.821 (0.787 - 0.854)**	**0.043 (0.015 - 0.072)**	0.783 (0.72 - 0.838)	0.041 (-0.013 - 0.092)	0.697 (0.666 - 0.726)	0.079 (0.046 - 0.112)
■	■	■	■	■		0.800 (0.762 - 0.836)	0.022 (-0.01 - 0.054)	0.793 (0.735 - 0.845)	0.051 (0.001 - 0.098)	**0.697 (0.668 - 0.727)**	**0.079 (0.046 - 0.113)**
■	■	■	■			0.806 (0.77 - 0.841)	0.028 (-0.004 - 0.06)	0.786 (0.727 - 0.841)	0.044 (-0.01 - 0.097)	0.634 (0.601 - 0.667)	0.017 (-0.014 - 0.048)
■	■		■			0.819 (0.784 - 0.851)	0.040 (0.011 - 0.069)	0.776 (0.716 - 0.831)	0.034 (-0.017 - 0.083)	0.632 (0.6 - 0.664)	0.015 (-0.015 - 0.045)
■	■	■				0.815 (0.778 - 0.849)	0.036 (0.007 - 0.066)	**0.802 (0.747 - 0.853)**	**0.060 (0.015 - 0.104)**	0.624 (0.59 - 0.657)	0.006 (-0.023 - 0.034)
■	■					0.810 (0.775 - 0.844)	0.032 (0.005 - 0.059)	0.798 (0.743 - 0.848)	0.056 (0.014 - 0.098)	0.624 (0.59 - 0.655)	0.006 (-0.023 - 0.034)
■						0.778 (0.74 - 0.814)		0.742 (0.688 - 0.793)		0.618 (0.586 - 0.649)	

Supplementary Table 10. Effect of feature types on AUPRC for prediction of tachycardia, hypotension, and hypoxia (120min window).

Area under the precision-recall curve (AUPRC) is evaluated on the test set. Each row represents a separately tuned and trained model. “Triage” variables include age, gender, triage vital signs, and chief complaint. “VS trend” denotes first vital signs from continuous monitoring, and the linear trends of each vital sign over a 15-minute assessment period. “PAT” denotes pulse arrival time, calculated from the ECG and PPG waveforms. “HRV” is a suite of heart rate variability measures. “Perfusion” is the perfusion index. “Waveform” indicates an 8-dimensional embedding generated from a transformer model, with 4 features each from the PPG and ECG waveforms.

Triage	VS Trends	PAT	HRV	Perfusion	Waveform	Tachycardia AUPRC	Hypotension AUPRC	Hypoxia AUPRC
■	■				■	0.261 (0.204 - 0.329)	0.176 (0.106 - 0.265)	0.255 (0.215 - 0.301)
■	■	■	■	■	■	0.267 (0.212 - 0.342)	0.165 (0.100 - 0.248)	0.274 (0.236 - 0.323)
■	■	■	■	■		0.280 (0.218 - 0.353)	0.167 (0.100 - 0.253)	0.275 (0.236 - 0.322)
■	■	■	■			0.251 (0.198 - 0.321)	0.182 (0.111 - 0.269)	0.238 (0.202 - 0.282)
■	■		■			0.263 (0.209 - 0.337)	0.165 (0.099 - 0.259)	0.241 (0.205 - 0.286)
■	■	■				0.268 (0.210 - 0.340)	0.174 (0.107 - 0.264)	0.241 (0.203 - 0.287)
■	■					0.250 (0.195 - 0.320)	0.173 (0.106 - 0.263)	0.231 (0.195 - 0.275)
■						0.213 (0.164 - 0.277)	0.110 (0.063 - 0.183)	0.198 (0.170 - 0.233)

Supplementary Table 11. Effect of feature types on AUROC for prediction of MEWS ≥ 4 (all prediction windows).

AUROC values and bootstrapped 95% CIs represent performance on the test set. Each row represents a separately tuned and trained model. “MEWS” is the Modified Early Warning Score. “Triage” variables include age, gender, triage vital signs, and chief complaint. “VS trend” denotes first vital signs from continuous monitoring, and the linear trends of each vital sign over a 15-minute assessment period. “PAT” denotes pulse arrival time, calculated from the ECG and PPG waveforms. “HRV” is a suite of heart rate variability measures. “Perfusion” is the perfusion index. “Waveform” indicates an 8-dimensional embedding generated from a transformer model, with 4 features each from the PPG and ECG waveforms. The best-performing model for each outcome is bolded.

Triage	VS trend	PAT	HRV	Perfusion	Waveform	60-minute window		90-minute window		120-minute window	
						AUROC (95% CI)	AUROC difference from triage-only model (95% CI)	AUROC (95% CI)	AUROC difference from triage-only model (95% CI)	AUROC (95% CI)	AUROC difference from triage-only model (95% CI)
■	■				■	0.819 (0.781 - 0.853)	0.058 (0.027 - 0.091)	0.824 (0.792 - 0.854)	0.051 (0.024 - 0.078)	0.808 (0.779 - 0.837)	0.045 (0.021 - 0.07)
■	■	■	■	■	■	0.819 (0.783 - 0.854)	0.059 (0.025 - 0.093)	0.817 (0.784 - 0.848)	0.044 (0.016 - 0.072)	0.811 (0.781 - 0.839)	0.048 (0.023 - 0.074)
■	■	■	■	■		0.817 (0.78 - 0.852)	0.056 (0.024 - 0.091)	0.816 (0.783 - 0.848)	0.044 (0.015 - 0.073)	0.814 (0.785 - 0.841)	0.051 (0.026 - 0.076)
■	■	■	■			0.817 (0.78 - 0.851)	0.056 (0.023 - 0.091)	**0.825 (0.794 - 0.856)**	**0.053 (0.027 - 0.079)**	0.812 (0.784 - 0.839)	0.049 (0.026 - 0.073)
■	■		■			0.815 (0.777 - 0.849)	0.054 (0.023 - 0.087)	0.820 (0.788 - 0.85)	0.047 (0.02 - 0.074)	**0.817 (0.789 - 0.845)**	**0.054 (0.032 - 0.078)**
■	■	■				**0.821 (0.785 - 0.854)**	**0.061 (0.030 - 0.093)**	0.823 (0.791 - 0.853)	0.050 (0.025 - 0.076)	0.815 (0.786 - 0.842)	0.052 (0.028 - 0.076)
■	■					0.821 (0.784 - 0.855)	0.061 (0.029 - 0.094)	0.818 (0.786 - 0.849)	0.045 (0.02 - 0.072)	0.806 (0.777 - 0.834)	0.043 (0.019 - 0.067)
■						0.761 (0.717 - 0.800)		0.773 (0.736 - 0.807)		0.763 (0.731 - 0.793)	

Supplementary Table 12. Effect of feature types on AUPRC for prediction of MEWS ≥ 4 (all prediction windows).

AUPRC values and bootstrapped 95% CIs represent performance on the test set. Each row represents a separately tuned and trained model. “MEWS” is the Modified Early Warning Score. “Triage” variables include age, gender, triage vital signs, and chief complaint. “VS trend” denotes first vital signs from continuous monitoring, and the linear trends of each vital sign over a 15-minute assessment period. “PAT” denotes pulse arrival time, calculated from the ECG and PPG waveforms. “HRV” is a suite of heart rate variability measures. “Perfusion” is the perfusion index. “Waveform” indicates an 8-dimensional embedding generated from a transformer model, with 4 features each from the PPG and ECG waveforms. The best-performing model for each outcome is bolded.

**Triage**	**VS trend**	**PAT**	**HRV**	**Perfusion**	**Waveform**	**60-minute window**		**90-minute window**		**120-minute window**	
						**AUPRC (95% CI)**	**AUPRC difference from triage-only model (95% CI)**	**AUPRC (95% CI)**	**AUPRC difference from triage-only model (95% CI)**	**AUPRC (95% CI)**	**AUPRC difference from triage-only model (95% CI)**
■	■				■	0.401 (0.326 - 0.48)	0.085 (0.028 - 0.142)	**0.437 (0.37 - 0.508)**	**0.104 (0.053 - 0.155)**	0.426 (0.367 - 0.491)	0.070 (0.030 - 0.116)
■	■	■	■	■	■	**0.403 (0.329 - 0.481)**	**0.087 (0.032 - 0.142)**	0.410 (0.343 - 0.481)	0.077 (0.031 - 0.125)	0.432 (0.372 - 0.495)	0.076 (0.031 - 0.124)
■	■	■	■	■		0.401 (0.327 - 0.479)	0.086 (0.03 - 0.141)	0.420 (0.353 - 0.489)	0.087 (0.040 - 0.133)	**0.445 (0.384 - 0.51)**	**0.089 (0.047 - 0.134)**
■	■	■	■			0.393 (0.319 - 0.472)	0.078 (0.022 - 0.134)	0.411 (0.346 - 0.481)	0.079 (0.033 - 0.124)	0.421 (0.361 - 0.483)	0.065 (0.025 - 0.107)
■	■		■			0.374 (0.302 - 0.452)	0.058 (0.006 - 0.111)	0.405 (0.34 - 0.475)	0.073 (0.025 - 0.121)	0.440 (0.381 - 0.502)	0.084 (0.043 - 0.126)
■	■	■				0.382 (0.309 - 0.464)	0.067 (0.007 - 0.13)	0.415 (0.349 - 0.485)	0.082 (0.036 - 0.128)	0.437 (0.378 - 0.502)	0.081 (0.040 - 0.127)
■	■					0.390 (0.316 - 0.470)	0.074 (0.021 - 0.129)	0.408 (0.342 - 0.481)	0.076 (0.029 - 0.125)	0.408 (0.351 - 0.473)	0.052 (0.012 - 0.098)
■						0.316 (0.248 - 0.390)		0.333 (0.272 - 0.399)		0.356 (0.301 - 0.416)	

Supplementary Table 13. Alignment between decompensation predictions and maximum MEWS≥4 in the test set (90min window).

For each individual abnormality predicted (tachycardia, hypotension, hypoxia), we produced dichotomous predictions of decompensation using operating points selected for 85% validation sensitivity. For visits predicted positive (+) or negative (-) for decompensation in the test set, we recorded the number of patients reaching MEWS≥4 during the prediction window. "Alignment" between predicted decompensation and MEWS is the proportion of patients reaching MEWS≥4 who were predicted to decompensate, or the proportion of patients with maximum MEWS<4 predicted not to decompensate.

	Triage-only			Best model		
**Tachycardia**		**MEWS ≥ 4**	**MEWS < 4**		**MEWS ≥ 4**	**MEWS < 4**
	**Predict +**	151	870	**Predict +**	140	567
	**Predict -**	69	1376	**Predict -**	80	1679
	**Total**	220	2246	**Total**	220	2246
	**Alignment**	68.6%	61.3%	**Alignment**	63.6%	74.8%
**Hypotension**		**MEWS ≥ 4**	**MEWS < 4**		**MEWS ≥ 4**	**MEWS < 4**
	**Predict +**	78	761	**Predict +**	67	763
	**Predict -**	142	1485	**Predict -**	153	1483
	**Total**	220	2246	**Total**	220	2246
	**Alignment**	35.5%	66.1%	**Alignment**	30.5%	66.0%
**Hypoxia**		**MEWS ≥ 4**	**MEWS < 4**		**MEWS ≥ 4**	**MEWS < 4**
	**Predict +**	186	1714	**Predict +**	177	1452
	**Predict -**	34	532	**Predict -**	43	794
	**Total**	220	2246	**Total**	220	2246
	**Alignment**	84.5%	23.7%	**Alignment**	80.5%	35.4%

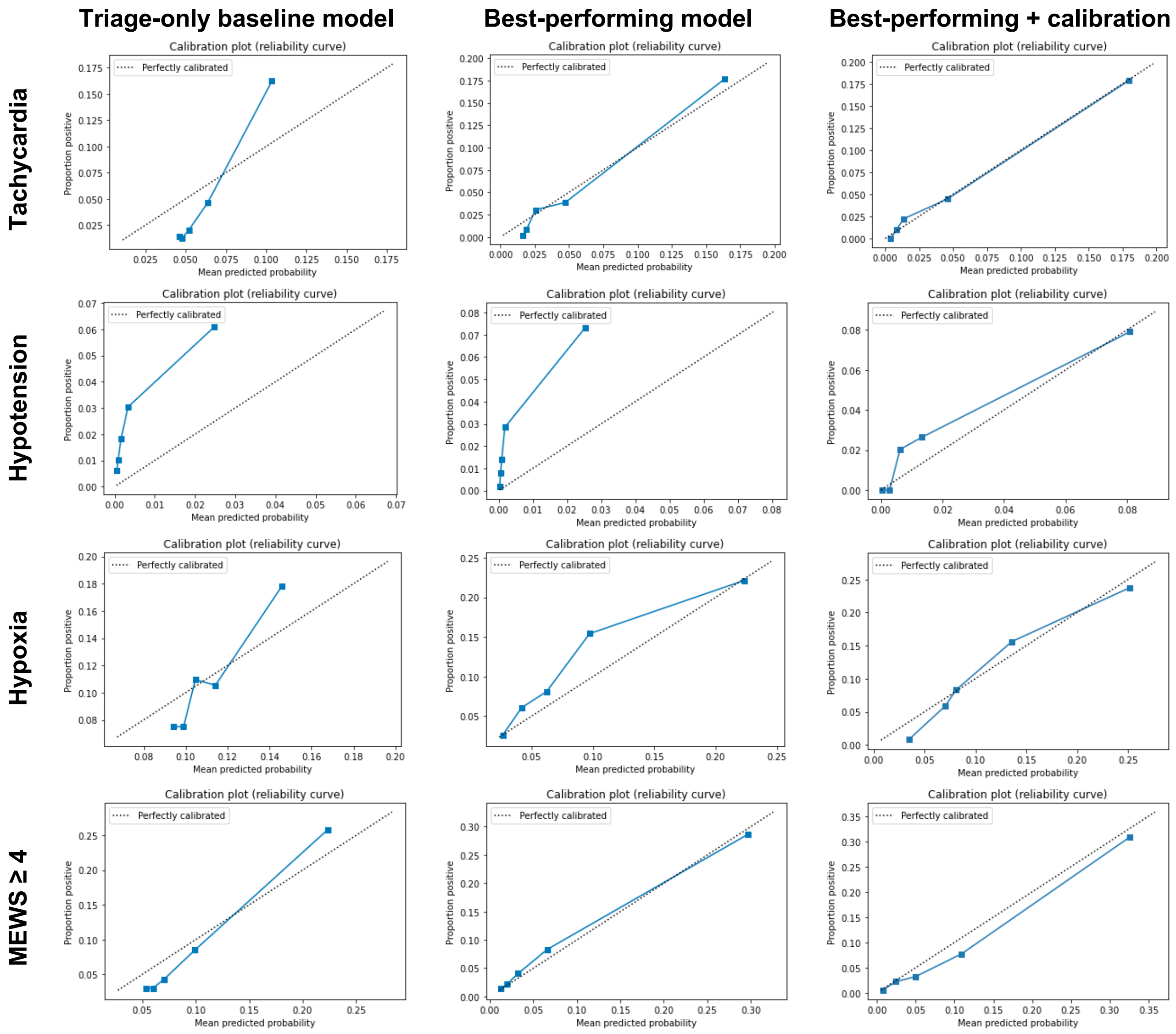

Supplementary Figure 1. Calibration plots for triage-only baseline and best-performing models for each outcome (90min window).

Right column shows best-performing models after application of isotonic regression (fit on validation set).

Supplementary Table 14. Contribution of feature types to model predictions (90min window).

Mean SHAP importance values were calculated for each feature in the baseline triage-only and full-feature models for all three tasks. Contribution (%) represents how much a given feature contributes to the model's prediction. Pearson's correlation coefficients represent the extent to which a higher feature value contributes to a positive prediction as assessed through SHAP analysis.

Tachycardia			Hypotension			Hypoxia		
**Top features**	**Contrib. (%)**	**Corr.**	**Top features**	**Contrib. (%)**	**Corr.**	**Top features**	**Contrib. (%)**	**Corr.**
Triage features only			Triage features only			Triage features only		
Triage HR	56.4	0.859	Triage SBP	22.1	-0.843	Age	20.4	0.876
Age	13.1	-0.773	Triage MAP	13.9	-0.693	Triage SpO2	19.5	-0.767
Triage temperature	6.6	0.617	Age	8.5	-0.257	Triage RR	10.8	0.667
Triage DBP	4.7	0.859	Triage DBP	8.0	-0.169	Triage SBP	9.4	-0.764
Triage RR	4.1	0.568	Triage temperature	7.2	-0.262	CC = Chest pain	6.5	-0.976
All features			All features			All features		
First mon. HR	19.5	0.941	First mon. MAP	13.0	-0.762	Perfusion index	23.2	-0.752
Trend HR	5.7	0.587	Triage SBP	8.4	-0.888	First mon. SpO2	17.1	-0.831
Triage HR	5.0	0.837	First mon. DBP	6.3	-0.472	Trend SpO2	6.7	-0.496
ECG waveform embedding 4	4.7	-0.943	First mon. SBP	5.8	-0.807	Triage RR	2.4	0.660
PPG waveform embedding 2	3.7	0.912	Trend SBP	3.2	-0.865	Trend SBP	2.4	0.625
Perfusion index	3.6	-0.832	Triage MAP	3.0	-0.715	Age	2.1	0.644
ECG waveform embedding 3	3.4	-0.666	Trend MAP	3.0	-0.765	Triage SpO2	1.9	-0.797
Age	3.1	-0.794	Perfusion index	3.0	-0.844	TINN	1.8	0.605
ECG waveform embedding 2	3.0	0.792	HF absolute power	2.8	0.449	ECG waveform embedding 3	1.7	-0.366
SDRR	2.8	0.865	First mon. RR	2.7	0.834	First mon. RR	1.5	0.596

Triage   First monitoring + trend   PAT   HRV   Perfusion index   Waveform

Supplementary Table 15. Differences in feature values among correctly reclassified visits (90min window).

We isolated the cohort that was reclassified by the best-performing model to either a correct or "true" positive or negative prediction over the baseline model. We characterized this reclassified cohort by identifying the top 10 most distinct features from the non-reclassified cohort. Differences were evaluated by two-sided t-tests.

Tachycardia

Top features		Cohort excluding reclassified correctly (mean +/- std)	Reclassified correctly to negative (mean +/- std) (n=545)	p-value of diff.	Top features		Cohort excluding reclassified correctly (mean +/- std)	Reclassified correctly to positive (mean +/- std) (n=6)	p-value of diff.
	Triage HR	80.51 (13.652)	89.774 (8.096)	<0.01		HF absolute power	0.01 (0.037)	0.003 (0.002)	<0.01
	Age	60.572 (20.396)	52.727 (19.985)	<0.01		pRR50	0.135 (0.206)	0.033 (0.035)	<0.01
	Triage DBP	79.139 (15.789)	83.333 (16.105)	<0.01		First monitor HR	78.851 (13.456)	94.796 (8.609)	<0.01
	PPG waveform embedding 4	6.44 (1.1)	6.638 (0.752)	<0.01		LF absolute power	0.025 (0.08)	0.010 (0.011)	0.015
	PPG waveform embedding 2	-1.098 (0.379)	-1.159 (0.259)	<0.01		Triage RR	17.986 (2.826)	14.714 (2.763)	0.027
	PPG waveform embedding 1	0.363 (0.324)	0.305 (0.292)	<0.01		Trend DBP	-0.148 (1.086)	-0.033 (0.096)	0.029
	ECG waveform embedding 1	0.013 (0.434)	-0.06 (0.384)	<0.01		ECG waveform embedding 3	-1.214 (0.244)	-1.453 (0.219)	0.037
	TINN	500.152 (302.294)	446.33 (268.362)	<0.01		ECG waveform embedding 4	4.509 (1.315)	3.178 (1.327)	0.049
	PPG waveform embedding 3	-0.847 (0.198)	-0.879 (0.18)	<0.01		First monitor DBP	82.526 (17.248)	96.714 (14.636)	0.055
	ECG waveform embedding 3	-1.214 (0.244)	-1.245 (0.2)	<0.01		PPG waveform embedding 2	-1.098 (0.379)	-0.731 (0.384)	0.058

Triage First monitoring + trend PAT HRV Perfusion index Waveform

Hypotension

	Top features	Cohort excluding reclassified correctly (mean +/- std)	Reclassified correctly to negative (mean +/- std) (n=189)	p-value of diff.		Top features	Cohort excluding reclassified correctly (mean +/- std)	Reclassified correctly to positive (mean +/- std) (n=15)	p-value of diff.
	Triage SBP	141.568 (24.366)	130.432 (18.993)	<0.01		First monitor SBP	140.0 (24.562)	126.375 (12.051)	0.02
	Triage MAP	100.5 (16.007)	96.06 (13.672)	<0.01		ECG waveform embedding 2	-0.581 (0.208)	-0.419 (0.143)	0.02
	First monitor RR	18.432 (4.545)	17.305 (4.333)	<0.01		PPG waveform embedding 1	0.03 (0.339)	0.34 (0.34)	0.047
	First monitor DBP	82.413 (17.339)	85.628 (14.041)	<0.01		First monitor SpO2	98.055 (1.862)	96.4 (2.264)	0.095
	ECG waveform embedding 2	-0.581 (0.208)	-0.539 (0.192)	<0.01		PPG waveform embedding 4	6.622 (0.23)	6.783 (0.224)	0.099
	ECG waveform embedding 4	7.105 (0.27)	7.143 (0.182)	<0.01		LF:HF ratio	3.884 (4.307)	2.932 (1.424)	0.123
	First monitor SBP	140.0 (24.562)	136.177 (19.372)	<0.01		PPG waveform embedding 2	-0.921 (0.31)	-0.643 (0.426)	0.127
	Age	59.75 (20.331)	56.032 (21.484)	<0.01		Triage SBP	141.568 (24.366)	133.625 (13.892)	0.175
	Trend SBP	-0.242 (0.952)	-0.097 (0.782)	<0.01		First monitor MAP	101.608 (17.002)	91.208 (18.894)	0.189
	HRVI	10.722 (6.645)	11.745 (6.536)	0.022		HF absolute peak	0.212 (0.055)	0.19 (0.042)	0.200

Triage First monitoring + trend PAT HRV Perfusion index Waveform

Hypoxia

	Top features	Cohort excluding reclassified correctly (mean +/- std)	Reclassified correctly to negative (mean +/- std) (n=540)	p-value of diff.		Top features	Cohort excluding reclassified correctly (mean +/- std)	Reclassified correctly to positive (mean +/- std) (n=24)	p-value of diff.
	Perfusion index	2.189 (1.835)	3.843 (1.901)	<0.01		Perfusion index	2.189 (1.835)	1.007 (1.151)	<0.01
	First monitor SpO2	97.933 (1.977)	98.61 (1.049)	<0.01		Triage SpO2	98.276 (1.891)	99.214 (1.013)	<0.01
	Triage SpO2	98.276 (1.891)	98.685 (1.388)	<0.01		HF absolute power	0.01 (0.039)	0.004 (0.007)	<0.01
	pRR50	0.144 (0.215)	0.094 (0.15)	<0.01		Age	59.683 (20.893)	46.679 (18.488)	<0.01
	RMSSD	58.119 (67.402)	43.005 (46.47)	<0.01		Acuity	2.693 (0.507)	2.964 (0.421)	<0.01
	First monitor HR	79.673 (12.862)	76.747 (12.157)	<0.01		LF absolute power	0.026 (0.079)	0.012 (0.023)	<0.01
	Trend SBP	-0.182 (0.947)	-0.381 (0.847)	<0.01		PAT	430.576 (168.177)	361.295 (150.447)	0.025
	TINN	502.006 (306.886)	441.242 (249.893)	<0.01		LF:HF ratio	3.94 (4.465)	3.037 (2.052)	0.034
	SDRR	57.59 (42.691)	50.176 (30.134)	<0.01		pRR50	0.144 (0.215)	0.092 (0.119)	0.053
	First monitor RR	18.48 (4.627)	17.65 (4.105)	<0.01		Trend RR	-0.023 (0.314)	-0.114 (0.235)	0.063

Triage   First monitoring + trend   PAT   HRV   Perfusion index   Waveform

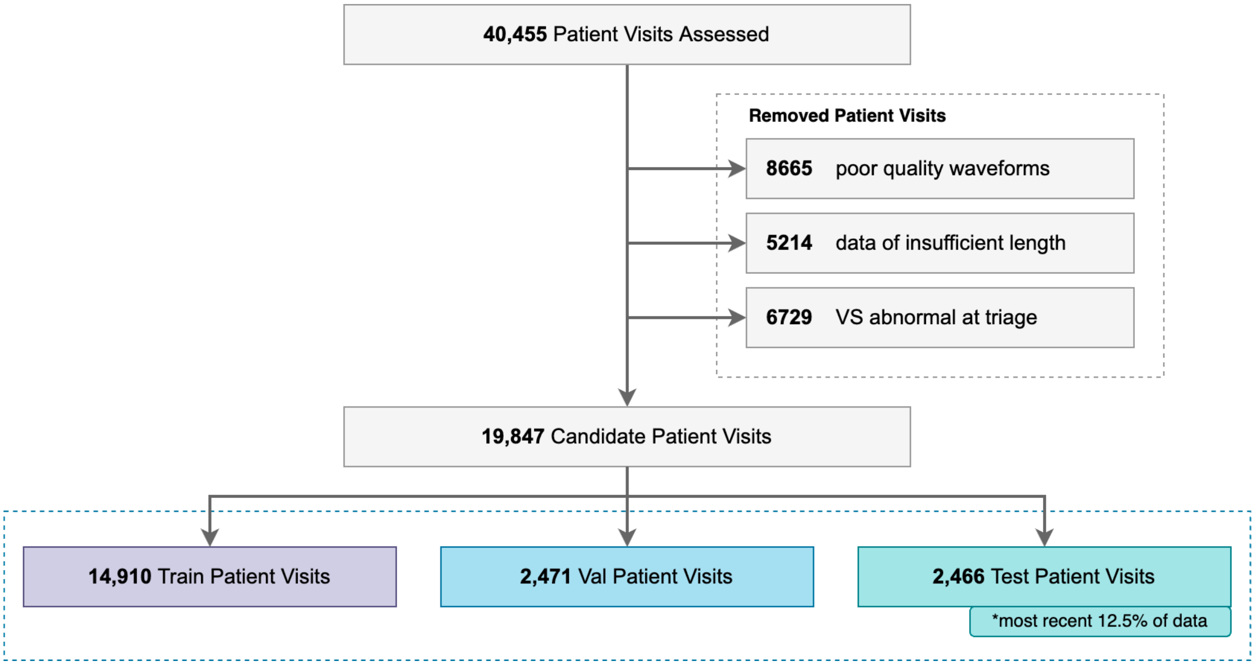

Supplementary Figure 2. Cohort creation.

We studied adult visits to monitored beds of the Stanford Health Care Emergency Department that occurred between August 1st 2020 and April 30th 2022. In order to predict new or unexpected decompensation, we included only visits with grossly normal vital signs at triage (HR≤110, SpO2≥90, MAP≥65), and excluded visits without at least one measurement of each vital sign and waveform. We divided the cohort into training (75%), validation (12.5%), and hold-out test sets (12.5%), with the test set containing visits occurring after those in the training and validation test sets, so as to simulate prospective validation.

Supplementary Table 16. Predictive performance of waveform transformer alone (90min window).

Primary models used transformer-derived embeddings as inputs to higher-level LGBM models. We also assessed the performance of the transformer alone in predicting decompensation.

Waveform input	Tachycardia AUROC	Hypotension AUROC	Hypoxia AUROC
ECG	0.746 (0.695 - 0.792)	0.561 (0.486 - 0.638)	0.543 (0.508 - 0.578)
PPG	0.783 (0.739 - 0.823)	0.462 (0.394 - 0.531)	0.593 (0.557 - 0.628)
ECG + PPG	0.785 (0.743 - 0.823)	0.525 (0.451 - 0.601)	0.598 (0.563 - 0.634)

Supplementary Table 17. Effect of waveform embedding length on AUROC for prediction of decompensation (90min window).

AUROC values are from the single, best-performing LGBM on the validation set, to assess the optimal waveform embedding length to use as input to the LGBM models. Performance on the validation set can exceed the test set performance reported elsewhere. Each row represents a separately tuned and trained model.

	Tachycardia AUROC (95% CI)	Hypotension AUROC (95% CI)	Hypoxia AUROC (95% CI)
**Triage**	0.798 (0.758 - 0.833)	0.798 (0.733 - 0.853)	0.605 (0.568 - 0.640)
**All features (4-dim ECG/PPG waveforms)**	0.868 (0.840 - 0.894)	0.859 (0.796 - 0.911)	0.706 (0.673 - 0.736)
**All features (8-dim ECG/PPG waveforms)**	0.862 (0.831 - 0.890)	0.839 (0.77 - 0.900)	0.711 (0.679 - 0.742)
**All features (16-dim ECG/PPG waveforms)**	0.867 (0.836 - 0.894)	0.842 (0.771 - 0.902)	0.701 (0.669 - 0.733)
**All features (32-dim ECG/PPG waveforms)**	0.861 (0.830 - 0.890)	0.826 (0.750 - 0.892)	0.703 (0.671 - 0.735)
**All features (64-dim ECG/PPG waveforms)**	0.872 (0.842 - 0.898)	0.837 (0.768 - 0.899)	0.706 (0.676 - 0.737)
**All features (128-dim ECG/PPG waveforms)**	0.864 (0.835 - 0.890)	0.843 (0.775 - 0.901)	0.699 (0.667 - 0.731)

Supplementary Table 18. Effect of waveform embedding length on AUPRC for prediction of decompensation (90min window).

AUPRC values are from the single, best-performing LGBM on the validation set, to assess the optimal waveform embedding length to use as input to the LGBM models. Performance on the validation set can exceed the test set performance reported elsewhere. Each row represents a separately tuned and trained model.

	**Tachycardia**<br>**AUPRC (95% CI)**	**Hypotension**<br>**AUPRC (95% CI)**	**Hypoxia**<br>**AUPRC (95% CI)**
**Triage**	0.231 (0.177 - 0.301)	0.071 (0.046 - 0.117)	0.169 (0.142 - 0.207)
**All features (4-dim ECG/PPG waveforms)**	0.327 (0.259 - 0.412)	0.174 (0.098 - 0.287)	0.238 (0.200 - 0.288)
**All features (8-dim ECG/PPG waveforms)**	0.319 (0.253 - 0.405)	0.161 (0.094 - 0.276)	0.255 (0.213 - 0.304)
**All features (16-dim ECG/PPG waveforms)**	0.353 (0.279 - 0.440)	0.145 (0.085 - 0.256)	0.246 (0.205 - 0.295)
**All features (32-dim ECG/PPG waveforms)**	0.351 (0.277 - 0.439)	0.132 (0.080 - 0.229)	0.252 (0.211 - 0.304)
**All features (64-dim ECG/PPG waveforms)**	0.335 (0.265 - 0.419)	0.173 (0.096 - 0.285)	0.242 (0.203 - 0.293)
**All features (128-dim ECG/PPG waveforms)**	0.325 (0.255 - 0.407)	0.142 (0.085 - 0.243)	0.253 (0.211 - 0.306)
