# Supplementary material for: Predicting patient decompensation from continuous physiologic monitoring in the emergency department
Source: NPJ Digit Med. 2023 Apr 4;6:60. doi: 10.1038/s41746-023-00803-0 (PMC10073111; doi:10.1038/s41746-023-00803-0)
Supplement: Supplementary file 2 — REPORTING SUMMARY [file 41746_2023_803_MOESM2_ESM.pdf]

nature portfolio

Corresponding author(s): David Kim MD PhD

Last updated by author(s): Jan 20, 2023

Reporting Summary

Nature Portfolio wishes to improve the reproducibility of the work that we publish. This form provides structure for consistency and transparency in reporting. For further information on Nature Portfolio policies, see our Editorial Policies and the Editorial Policy Checklist.

Statistics

For all statistical analyses, confirm that the following items are present in the figure legend, table legend, main text, or Methods section.

n/a	Confirmed	
☐	☒	The exact sample size (*n*) for each experimental group/condition, given as a discrete number and unit of measurement
☐	☒	A statement on whether measurements were taken from distinct samples or whether the same sample was measured repeatedly
☐	☒	The statistical test(s) used AND whether they are one- or two-sided<br>*Only common tests should be described solely by name; describe more complex techniques in the Methods section.*
☐	☒	A description of all covariates tested
☐	☒	A description of any assumptions or corrections, such as tests of normality and adjustment for multiple comparisons
☐	☒	A full description of the statistical parameters including central tendency (e.g. means) or other basic estimates (e.g. regression coefficient) AND variation (e.g. standard deviation) or associated estimates of uncertainty (e.g. confidence intervals)
☐	☒	For null hypothesis testing, the test statistic (e.g. *F*, *t*, *r*) with confidence intervals, effect sizes, degrees of freedom and *P* value noted<br>*Give P values as exact values whenever suitable.*
☒	☐	For Bayesian analysis, information on the choice of priors and Markov chain Monte Carlo settings
☒	☐	For hierarchical and complex designs, identification of the appropriate level for tests and full reporting of outcomes
☐	☒	Estimates of effect sizes (e.g. Cohen's *d*, Pearson's *r*), indicating how they were calculated

Our web collection on statistics for biologists contains articles on many of the points above.

Software and code

Policy information about availability of computer code

Data collection	Continuous monitor data was extracted from the Stanford Health Care Philips Data Warehouse using Philips PIC iX DWC Toolkit C.03.31
Data analysis	All analyses were performed using Python (3.9.7). Data processing was performed using numpy (1.21.6), pandas (1.4.2), h5py (3.6.0) and scikit-learn (1.0.1). Cohort statistical analysis was performed using scipy (1.8.0). HRV/PTT feature extraction was performed using scipy (see above) and matplotlib (3.5.1). Transformer training and evaluation was performed using torch (1.10.2+cu111), pytorch_lightning (1.6.1), torchmetrics (0.8.0), edm (0.0.4) and wandb (0.12.14). Additionally, the edm package uses biosppy (0.6.1) and vital-sqi (0.1.0). LGBM model training, tuning and evaluation was performed using lightgbm (3.3.0), scikit-learn (see above) and verstack (3.2.3). Secondary analyses of model performance were performed using shap (0.40.0), scikit-learn (see above), matplotlib (see above) and scipy (see above).

For manuscripts utilizing custom algorithms or software that are central to the research but not yet described in published literature, software must be made available to editors and reviewers. We strongly encourage code deposition in a community repository (e.g. GitHub). See the Nature Portfolio guidelines for submitting code & software for further information.

Data

Policy information about availability of data

All manuscripts must include a data availability statement. This statement should provide the following information, where applicable:

- Accession codes, unique identifiers, or web links for publicly available datasets
- A description of any restrictions on data availability
- For clinical datasets or third party data, please ensure that the statement adheres to our policy

A de-identified dataset sufficient to reproduce main results is available from the corresponding author upon reasonable request. The original study dataset contains protected health information and cannot be distributed.

Human research participants

Policy information about studies involving human research participants and Sex and Gender in Research.

Reporting on sex and gender	We use patient self-reported gender (as reflected in the study site's electronic health record) in all analyses reporting gender.
Population characteristics	The study population is described in detail in Table 1.
Recruitment	The study dataset is retrospective and derived from complete records of all monitored adult ED visits during the study period.
Ethics oversight	Stanford University Institutional Review Board.

Note that full information on the approval of the study protocol must also be provided in the manuscript.

Field-specific reporting

Please select the one below that is the best fit for your research. If you are not sure, read the appropriate sections before making your selection.

☒ Life sciences ☐ Behavioural & social sciences ☐ Ecological, evolutionary & environmental sciences

For a reference copy of the document with all sections, see nature.com/documents/nr-reporting-summary-flat.pdf

Life sciences study design

All studies must disclose on these points even when the disclosure is negative.

Sample size	The sample is composed of all adult ED patients with continuous monitor data, at the time of study design and analysis.
Data exclusions	We excluded visits without at least one measurement of each vital sign and waveform.
Replication	We have replicated all results by applying the code in the public repository directly to the study data.
Randomization	N/A, no randomization/intervention.
Blinding	N/A, no randomization/intervention.

Reporting for specific materials, systems and methods

We require information from authors about some types of materials, experimental systems and methods used in many studies. Here, indicate whether each material, system or method listed is relevant to your study. If you are not sure if a list item applies to your research, read the appropriate section before selecting a response.

Materials & experimental systems

n/a	Involved in the study
☒	☐ Antibodies
☒	☐ Eukaryotic cell lines
☒	☐ Palaeontology and archaeology
☒	☐ Animals and other organisms
☒	☐ Clinical data
☒	☐ Dual use research of concern

Methods

n/a	Involved in the study
☒	☐ ChIP-seq
☒	☐ Flow cytometry
☒	☐ MRI-based neuroimaging
